# Genome‐wide analysis of natural and restored eastern oyster populations reveals local adaptation and positive impacts of planting frequency and broodstock number

**DOI:** 10.1111/eva.13322

**Published:** 2021-12-07

**Authors:** Katherine M. Hornick, Louis V. Plough

**Affiliations:** ^1^ University of Maryland Center for Environmental Science Horn Point Laboratory Cambridge Maryland USA

**Keywords:** aquaculture, genomics, hatchery, local adaptation, oyster, RADseq, restoration

## Abstract

The release of captive‐bred plants and animals has increased worldwide to augment declining species. However, insufficient attention has been given to understanding how neutral and adaptive genetic variation are partitioned within and among proximal natural populations, and the patterns and drivers of gene flow over small spatial scales, which can be important for restoration success. A seascape genomics approach was used to investigate population structure, local adaptation, and the extent to which environmental gradients influence genetic variation among natural and restored populations of Chesapeake Bay eastern oysters *Crassostrea virginica*. We also investigated the impact of hatchery practices on neutral genetic diversity of restored reefs and quantified the broader genetic impacts of large‐scale hatchery‐based bivalve restoration. Restored reefs showed similar levels of diversity as natural reefs, and striking relationships were found between planting frequency and broodstock numbers and genetic diversity metrics (effective population size and relatedness), suggesting that hatchery practices can have a major impact on diversity. Despite long‐term restoration activities, haphazard historical translocations, and high dispersal potential of larvae that could homogenize allele frequencies among populations, moderate neutral population genetic structure was uncovered. Moreover, environmental factors, namely salinity, pH, and temperature, play a major role in the distribution of neutral and adaptive genetic variation. For marine invertebrates in heterogeneous seascapes, collecting broodstock from large populations experiencing similar environments to candidate sites may provide the most appropriate sources for restoration and ensure population resilience in the face of rapid environmental change. This is one of a few studies to demonstrate empirically that hatchery practices have a major impact on the retention of genetic diversity. Overall, these results contribute to the growing body of evidence for fine‐scale genetic structure and local adaptation in broadcast‐spawning marine species and provide novel information for the management of an important fisheries resource.

## 1 INTRODUCTION

Anthropogenic impacts to aquatic environments, including habitat deterioration, species introductions, and overharvesting, have degraded ecosystems and reduced populations of species worldwide, with coastal marine environments among the most severely affected (Lotze et al., 2006). To counteract these impacts, reestablish ecosystem function, and build resiliency, restoration activities, including population supplementation with translocated stock from natural populations and captive‐reared offspring, have become important fisheries management strategies (see Bell et al., 2008 for definitions and objectives, Lorenzen et al., 2012). While these activities have increased population abundances of target species (e.g., Berejikian & Doornik, 2018), they may also have profound evolutionary impacts that can reduce long‐term population fitness and resilience (reviewed in Frankham et al., 2002). Therefore, understanding patterns of neutral and adaptive genetic variation is critical to establishing restoration programs that aim to preserve genetic diversity, maintain historic gene flow and local adaptation, and promote resilience in the face of rapid environmental change (Flanagan et al., 2018; Laikre et al., 2010). While recent advances in genomics allow more precise quantification of neutral variation and the identification of adaptive loci affected by the environment (e.g., Allendorf et al., 2010; Baird et al., 2008), more work is needed to link these approaches with practical aspects of species restoration (e.g., Breed et al., 2018).

Key issues for many restoration programs are the degree to which genetic diversity is maintained in hatchery‐produced individuals compared with natural populations and the choice of appropriate broodstock material (Broadhurst et al., 2008). Restoration with hatchery‐produced individuals can have profound and rapid effects on the genetic composition and diversity of receiving populations, in many cases negatively impacting population viability and resilience (reviewed in Frankham et al., 2002). For example, reductions in genetic diversity and effective population size (*N*_e_, the evolutionary analog to census population size) have been documented in supplemented populations when large numbers of hatchery‐produced individuals from a small number of broodstock are released (Christie et al., 2012; Ryman et al., 1995; Ryman & Laikre, 1991). Transplanting foreign genotypes with lower fitness than local genotypes can have important implications for restoration success and the long‐term viability of restored populations (Galloway & Fenster, 2000; Helenurm, 2008; Hufford & Mazer, 2003; Montalvo & Ellstrand, 2001). Restoration guidelines advocate the use of local, wild broodstock (e.g., Brumbaugh et al., 2006), but these guidelines often assume high connectivity and minimal population structure among populations of marine species with planktonic dispersal. However, recent studies of marine species indicate both limited effective dispersal and local adaptation over small scales may be more common than previously hypothesized (e.g., Bernatchez et al., 2019; Hauser & Carvalho, 2008; Sanford & Kelly, 2011; Silliman, 2019). Therefore, the choice of appropriate genetic material for population restoration programs requires an understanding of population structure and patterns of adaptation across a broad range of environmental scales. While “genetically aware” restoration programs exist (i.e., broodstock are selected from local populations and carefully planned breeding protocols are utilized), the severity of associated genetic changes remains variable (e.g., Christie et al., 2012; Gow et al., 2011; Heggenes et al., 2006). Furthermore, understanding how captive breeding impacts genetic diversity of restored populations has been studied intensively in only a few species of finfish (e.g., Berejikian & Van Doornik, 2018; Christie et al., 2012; Hagen et al., 2021) and less work has been conducted in other exploited marine species such as shellfish.

Restoration of marine bivalve populations has become commonplace across the USA and is gaining momentum worldwide, largely in response to widespread population decline (Beck et al., 2011; Fariñas‐Franco et al., 2018; Pogoda, 2019) and an increasing appreciation of the ecosystem services that healthy reefs provide (Smaal et al., 2019; zu Ermgassen et al., 2020). Bivalve restoration often includes supplementing natural populations with hatchery‐propagated juveniles (Carranza & zu Ermgassen, 2020; Gaffney, 2006; Laing et al., 2006). While associated genetic impacts resulting from hatchery propagation have been documented (Boudry et al., 2002; Camara & Vadopalas, 2009; Lind et al., 2009; Lallias et al., 2010, Varney & Wilbur, 2020), relatively few studies have assessed how hatchery supplementation and production techniques may impact genetic diversity of restored reefs (Arnaldi et al., 2018; Hornick & Plough, 2019; Hughes et al., 2019; Jaris et al., 2019; Morvezen et al., 2016). Patterns of neutral and adaptive genetic variation in natural populations of bivalves have been uncovered in recent studies using high‐resolution genomic methods (Bernatchez et al., 2019; Lehnert et al., 2019; Miller et al., 2019; Silliman, 2019; Vendrami et al., 2019). Marine bivalves exhibit complex life‐history features such as high‐fecundity, type‐III survivorship, and high variance in reproductive success (e.g., Hedgecock & Pudovkin, 2011; Plough, 2016; Plough et al., 2016), which can reduce *N*_e_ and genetic diversity in hatchery‐produced juveniles and exacerbate the negative genetic impacts associated with restoration. While genetic information is frequently integrated in terrestrial ecosystem restoration planning (Leimu & Fischer, 2008; McKay et al., 2005; Rice & Emery, 2003), it is considered yet rarely integrated into marine restoration planning (Baums, 2008; for exceptions, see Camara & Vadopalas, 2009; Fraser et al., 2011; Hämmerli & Reusch, 2002).

In this study, next‐generation sequencing and a more expansive sampling of restored and natural reefs than previous studies (e.g., Hornick & Plough, 2019) were used to examine the genetic impact of a large‐scale hatchery‐based restoration program for eastern oysters in the Chesapeake Bay. Contemporary Chesapeake Bay oyster populations have declined to ~1% of historic abundances (Mackenzie, 2007; Wilberg et al., 2011); thus, a variety of management and restoration efforts have been undertaken, including seed translocations within and between Bay tributaries, the construction of reef habitat using fresh and dredged shell, designation of oyster sanctuaries or reserves, and supplementing reefs with hatchery‐produced juveniles or large adults (Brumbaugh & Coen, 2009; Coen & Luckenbach, 2000; Kennedy & Breisch, 1983). The Chesapeake Bay region has a long history of oyster restoration, and recent strategies are based on information gained over many decades of restoration planning and management (e.g., Kennedy et al., 2011), including the extensive consideration of oyster genetics (e.g., Allen & Hilbish, 2000; USACE, 2009, 2012). A federal mandate to restore 20 Chesapeake Bay tributaries by 2025 has provided support for large‐scale restoration in the Choptank River (Maryland, USA), with the first sanctuary, Harris Creek, completed in 2016 (Westby et al., 2017). The University of Maryland Center for Environmental Science's (UMCES) Horn Point Laboratory (HPL) Oyster Hatchery produces spat (juvenile oysters) for Harris Creek (and other tributaries), through mass‐spawning of local, natural broodstock. While initial characterization of the neutral genetic impacts of this program has been conducted (Hornick & Plough, 2019), the analysis of additional natural and restored populations using high‐resolution genome‐wide markers is necessary to infer patterns of neutral and adaptive genetic variation of Chesapeake Bay oyster populations. This information will permit a more complete understanding of the genetic impacts of large‐scale hatchery‐based oyster restoration.

Here, we characterized patterns of genetic variation within and among natural and restored eastern oyster populations to quantify the broader population genetic impacts of large‐scale hatchery‐based bivalve restoration, investigate population structure, local adaptation, and the extent at which environmental gradients influence genetic variation among these populations. This is the first study to include fine‐scale sampling of restored bivalve populations with variable hatchery‐planting efforts and to utilize thousands of high‐resolution single nucleotide polymorphisms (SNPs) to characterize neutral and adaptive genetic variation and structure of restored and wild oyster populations in the Chesapeake Bay. Understanding the extent of genetic variability in natural and restored oyster populations and how the variation is structured across broad environmental gradients could provide important information for planning future bivalve restoration programs and their management.

## 2 MATERIALS AND METHODS

### 2.1 Sample collection

**FIGURE 1:**
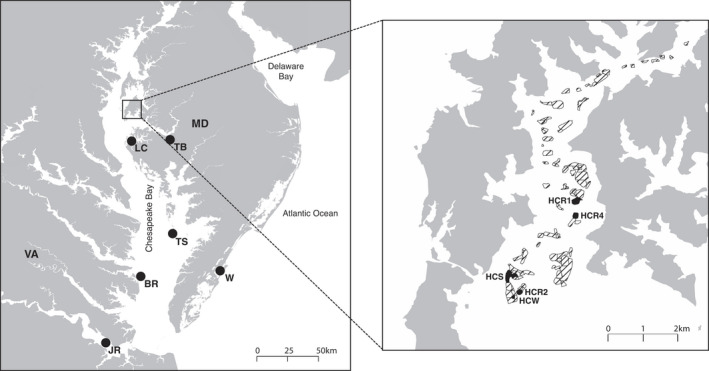
Map of sampling locations of eastern oysters within the Chesapeake Bay. Abbreviations of sampling sites are presented in Table 1

Oysters were collected between 2015 and 2018 from nine sites throughout the Chesapeake Bay (Table 1 and Figure 1). For the Harris Creek sites, divers sampled putative wild natural oysters (based on sampling location and reef characteristics), recently recruited juveniles (spat), and adult oysters from sites with variable hatchery‐planting efforts (Table 1 and Figure 1). Samples collected from restored reefs in Harris Creek included sites planted with hatchery oysters during one season, two seasons, and four seasons (a season occurs during the summer/fall and may involve more than one hatchery‐planting event) to assess genetic changes associated with planting frequency. For the natural Maryland populations, oysters were obtained from the Choptank River hatchery broodstock source population, States Bank (Figure 1). Natural Virginia populations included oysters from sites with no previous hatchery‐produced restoration plantings at the scale of the program in Harris Creek (tens of millions of seeds planted each year). All samples represent mixed‐age cohorts (see Table 1 for average length of oysters from each site), except the recently recruited spat sample from Harris Creek (HCS). Tissues were sampled from adductor muscle or mantle and preserved in 70–95% ethanol until DNA extraction (*N* = 556 individuals).

**TABLE 1:** Location, latitudinal range, type, sample size, number of samples successfully genotyped (*N*_gen_), size ranges, and station ID from which environmental data was used for redundancy analysis for each Chesapeake Bay sampling site of eastern oysters. Hatchery plantings denote the number of seasons a restored site was planted with hatchery‐produced oysters

Site	Abbreviation	Type	Hatchery plantings	Latitude	Longitude	Location	*N*	*N*_gen_	Size (mm)	Station ID
Harris Creek	HCR1	Restored	2014	38.735323	−76.30243	MD	50	33	103.7 ± 17.7	XFG4618
Harris Creek	HCR2	Restored	2015, 2016	38.711485	−76.316936	MD	50	37	57.3± 8.8	XFG2810
Harris Creek	HCR4	Restored	2011, 2013, 2014, 2017	38.731909	−76.302688	MD	50	43	67.4± 1.9	XFG4618
Harris Creek	HCS		2015, 2016	38.715637	−76.320025	MD	60	49	––	XFG2810
Harris Creek	HCW	Natural		38.710212	−76.318738	MD	60	53	––	XFG2810
Little Choptank	LC	Natural		38.5368	−76.254303	MD	48	47	87.4± 10.9	EE2.2
States Bank	TB	Natural		38.57	−76.04	MD	48	48	127.3 ± 23.5	ET5.2
Beverly's Rock	BR	Natural		37.5322	−76.253	VA	50	48	45–95	CB5.3
James River	JR	Natural		37.012	−76.468	VA	48	47	75.2 ± 16.3	LE5.3
Tangier Sound	TS	Natural		37.78303	−75.94814	VA	50	38	62.6 ± 13.3	EE3.2
Wachapreague	W	Natural		37.612233	−75.66795	VA	50	35	102.7 ± 24.2	XBM8828

### 2.2 Library preparation and bioinformatics

Double digest restriction‐site‐associated DNA (ddRAD) (Peterson et al., 2012) libraries were prepared and sequenced on two and a half lanes of the Illumina HiSeq 2500 platform at Genewiz, Inc. (South Plainfield, NJ). Two sampling sites were spread across libraries to minimize batch effects from library preparation, and sequencing of 14 individuals in duplicate was included to estimate sequencing and genotyping errors. Demultiplexing was performed using the *process_radtags* component of Stacks v.2.0 (Catchen et al., 2013), and read mapping and SNP calling were performed using the dDocent pipeline v. 2.7.7 (Puritz et al., 2014) with default settings unless otherwise noted. Trimmed reads were mapped to the latest release of the *C*. *virginica* genome (NCBI Bioprojects: PRJNA379157 and PRJNA376014, accession numbers: NC_035780.1–NC_035789.1). Freebayes v1.2.0‐dirty (Garrison & Marth, 2012) was used to obtain raw variant calls and SNP genotypes. Additional details of the above procedures are given in Supplementary Methods S1 and S2.

### 2.3 Outlier detection and defining datasets

To account for false positives in outlier detection, the identification of SNPs putatively under selection was assessed by using three outlier detection methods with different underlying models as recommended by Hoban et al. (2016): Bayescan (v.2.1) (Foll & Gaggiotti, 2008), OutFLANK (v.0.2) (Whitlock & Lotterhos, 2015), and pcadapt (v.4.1.0). For these analyses, comparisons were made among populations grouped by sampling site. SNPs with a global, major‐allele frequency above 0.95 were excluded from all outlier detection approaches because low minor allele frequencies can bias results (Roesti et al., 2012). Additional details of outlier detection methods are given in Supplementary Methods S3.

The dataset was subdivided into “neutral” and “outlier” components with the final outlier dataset consisting of all SNPs identified as outliers under directional selection by at least one of the approaches, and all outliers detected in the redundancy analysis (RDA; details below); the neutral dataset consisted of all remaining SNPs. Any SNP identified as an outlier in at least one method was removed from the neutral dataset. SNPs that were detected as outliers using all methods constituted the putatively adaptive dataset. All outlier analyses were repeated using the same criteria excluding the Virginia coastal Bay Wachapreague sample to test for selection within Chesapeake Bay samples (inner Bay dataset).

Inclusion of loci that are strongly linked (high linkage disequilibrium) can lead to biases in downstream analyses if independence of loci is assumed (Willis et al., 2017). For inferences of genetic diversity and population structure, a dataset was created that excluded SNPs in close proximity in the genome. Thinning of the neutral dataset by chromosome was performed in VCFTOOLS using the thin function (Danecek et al., 2011). The appropriate thinning distance was determined by calculating *R* ^2^ separately for SNPs on the same chromosome (intrachromosomal pairs) and for unlinked SNPs (interchromosomal pairs). The critical *R* ^2^ was estimated from the unlinked loci by root transforming the *R* ^2^ values and taking the 95th percentile of the distribution as the threshold beyond which the LD is caused by physical linkage (Breseghello & Sorrells, 2006). The relationship between LD decay and genetic distance was summarized by fitting a second‐degree smoothed locally weighted linear regression (LOESS) curve (Cleveland, 1979) to intrachromosomal *R* ^2^ data in R. The distance the loess curve intercepted the critical R^2^ was identified as the threshold for LD decay (Figure S1).

### 2.4 Genetic diversity and effective population size of natural and restored oysters

The thinned SNP dataset (4641 SNPs) was used to calculate observed (*H*_o_) and expected (*H*_e_) heterozygosity, allelic richness (A_r_), and the *F*_IS_ inbreeding coefficient in hierfstat v0.04‐22 (Goudet, 2005; R Core Team, 2020). Confidence intervals for population‐specific *F*_IS_ were determined using the *boot*.*ppfis* function in hierfstat with 1000 bootstrap replicates. Relatedness was estimated for natural and restored oysters using the R package related v.0.8 (Pew et al., 2015). The Ritland estimator (Ritland, 1996) was used because it has been shown to have the least bias with small sample sizes (Wang, 2017).

Contemporary genetic effective population size (*N*_e_) was estimated using the single‐sample linkage disequilibrium method (Hill, 1981; Waples, 2006; Waples & Do, 2010) as implemented in NeEstimator v2.1 (Do et al., 2014) under a random mating model. The Harris Creek spat sample (which represents a single cohort) provides information most relevant to estimating the number of breeders (*N*_b_; Waples, 2005), while the mixed‐age cohorts (all remaining sites) provide information relevant to estimating *N*_e_ (Waples et al., 2014). The neutral dataset was used for *N*_e_ estimation (i.e., excluding loci putatively under selection) as suggested by Waples (2006), and P_Crit_ was set to 0.02 (alleles with frequencies <0.02 are excluded), which balances effects of precision and bias (Waples & Do, 2010). Confidence intervals were based on the jackknife method (Jones et al., 2016). While the spat sample provides information relevant to estimating *N*_b_, there is some influence from background *N*_e_ per generation (Waples et al., 2014). To overcome bias due to overlapping generations using the LD *N*_e_ method, the raw *N*_b_ estimate from the Harris Creek spat sample was adjusted according to Waples et al. (2014) using three life‐history traits as in Hornick and Plough (2019): adult life span = 15 (10–20 years in undisturbed populations, Powell & Cummins, 1985), age at maturity (*α*) = 2 (averaged values from Galtsoff, 1964; Powell et al., 2013; Rothschild et al., 1994), and variation in age‐specific fecundity CVf  = 0.65 (from Mann et al., 2014; Mroch et al., 2012).

The association between reef size, number of broodstock, male‐to‐female ratio of broodstock, and planting frequency and genetic diversity metrics of restored reefs was investigated (mixed‐cohort samples) using generalized linear models. The association between *N*_e_ and *H*_o_ at restored reefs to planting frequency, number of broodstock used each planting season, male‐to‐female ratio of broodstock used each planting season, and reef size (acres; Table S1) was examined. For this analysis, data from a restored reef in Harris Creek, which was planted with hatchery‐produced oysters in 2012 and genotyped with nine microsatellite markers, were included (Hornick & Plough, 2019). To ensure that heterozygosity of all individuals was measured on the same scale despite differences in marker information from SNPs versus microsatellite markers, the standardized multilocus heterozygosity, the sum of observed average heterozygosity in a population (Coltman et al., 1999), was calculated using the R package inbreedR v.0.3.2 (Stoffel et al., 2016). Significant correlations between the predictors and genetic diversity metrics of restored reefs were calculated in R.

### 2.5 Genetic differentiation, population structure, and population assignment

All analyses related to neutral population genetic structure were performed using the thinned, neutral dataset. The extent of genetic differentiation between the sampling sites was evaluated using pairwise estimates of *F*_ST_ (Weir & Cockerham, 1984) with the *genet*.*dist* function in hierfstat. Isolation by distance (IBD, Sokal, 1979) was evaluated using a Mantel test of pairwise *F*_ST_ values coded as *F*_ST_/(1–*F*_ST_) as a function of water distance between sampling sites (calculated by drawing routes between all sites on Google Earth) as implemented in adegenet v.2.1.1 (Jombart, 2008; Jombart & Ahmed, 2011).

Two approaches were used to investigate neutral spatial genetic structure: the multivariate discriminant analysis of principal components (DAPC) and the Bayesian clustering algorithm implemented in STRUCTURE v.2.3.4 (Pritchard et al., 2000). Clustering identification was performed by cross‐validated DAPC implemented in the r package adegenet (Jombart, 2008; Jombart & Ahmed, 2011). Individuals were grouped based on sampling site. Cross‐validation was performed over a range of 1–478 PCs with 500 replicates to determine the number of principal components to retain and to avoid overfitting during discrimination. After the number of optimal PCs was identified, a second cross‐validation was performed for a narrower range of principal components (±10 of the previously identified optimum). Membership of individuals to clusters was defined by independent k‐means, using the Bayesian information criterion (BIC). Next, the Bayesian clustering method STRUCTURE v.2.3.4 (Pritchard et al., 2000) was used to identify the number of distinct genetic clusters (K) with a burn‐in of 50,000 iterations followed by an additional 200,000 Markov chain Monte Carlo (MCMC) steps, using prior sampling location information and the no‐admixture model, which is preferred when levels of divergence between populations are low (Hubisz et al., 2009). Fifteen replicates of K from 1 to 11 were performed, where K is the number of population clusters. Replicates were summarized and visualized using the CLUMPAK server (Kopelman et al., 2015). The K method in STRUCTURE HARVESTER was used to determine the optimal K (Earl & vonHoldt, 2012).

### 2.6 Genotype–environment associations

A RDA was performed as a genotype–environment association method to detect SNPs putatively under selection based on correlations with environmental variables as described in Capblancq et al. (2018) using the R package vegan v.2.5–5 (Oksanen, 2017). Environmental data for each locality was obtained from the Maryland Department of Natural Resources Eyes on the Bay program (http://eyesonthebay.dnr.maryland.gov/) and the Chesapeake Bay Program (http://data.chesapeakebay.net/) from buoys located closest to each of the eleven sampling sites (Table 1). Variance inflation factors (VIF) were calculated to check for multicollinearity, and variables were retained if their VIF was <10. Statistical significance (alpha ≤ 0.05) of the model and of each axis was tested used a permutation‐based analysis of variance (999 permutations). Following the constrained ordination step, outlier SNPs were detected using the pcadapt methodology (Capblancq et al., 2018; Luu et al., 2017). After visual inspection of the amount of information retained on the different axes of RDA, only *z*‐scores of the two most significant ordination axes were retained for subsequent analysis. For each SNP, a robust Mahalanobis distance was computed to identify outlier vectors of *z*‐scores (Capblancq et al., 2018) using the R package robust v.0.4‐18.2 (Wang et al., 2019). A false discovery rate (FDR) approach was used to control for false positives, with markers having *q*‐values less than 0.1 considered as significantly associated with environmental gradients. Each SNP was assigned to the environmental predictor for which the correlation was the highest (Forester et al., 2018, see https://popgen.nescent.org/2018‐03‐27_RDA_GEA.html for details).

### 2.7 Effect of environmental variables and geography on genetic variation

Redundancy analysis was conducted on the neutral and putative outlier datasets separately to assess the influence of environmental variables and geographic distance on observed patterns of genetic variation (Bie et al., 2012; Borcard et al., 1992; Legendre & Fortin, 2010; Liu, 1997). Significance of components of genetic variance explained by geography, environment, and the interaction between the two was tested using 1000 permutations. To explain how much of the genetic variation in *C*. *virginica* is uniquely explained by environmental variables, how much is uniquely explained by geography, and how much is due to the combined effect of the two, variance components of the RDA were partitioned by running 3 models: a full model with environmental and geographic variables, a partial model in which geography explains genetic data conditioned on important environmental variables, and a partial model in which important environmental variables explain genetic data conditioned on geography. This analysis allowed for distinguishing between how much of the total explainable neutral and adaptive variance was due to the environment (after removing geographical effects), how much was due to geography (after removing environmental effects), and how much was due to the joint effect of both factors. Additional details of this approach are given in Supplementary Methods S5.

### 2.8 Functional annotation of outlier loci

To gain insight into possible targets of selection, we performed a gene ontology (GO) annotation of SNPs identified as outliers in at least two differentiation‐based outlier detection methods and identified in RDA. The resulting flanking regions (100 bp) of these SNPs were extracted from the eastern oyster genome that we previously used for the bioinformatics pipeline and BLASTed (Altschul et al., 1990; minimum *e*‐value of 0.001) on the protein sequences of *C*. *virginica*. We used GO terms generated in Johnson and Kelly (2020). For variants that resulted in the same protein result, we evaluated whether the amino acid sequence was the same or not. If amino acid sequences were different, we conducted a search on the SWISS‐PROT database (Bairoch & Apweiler, 2000) using the protein name.

## 3 RESULTS

### 3.1 Outlier detection and defining datasets

The full, final dataset consisted of 6654 SNPs from 478 individuals (summary of data filtering is presented in Table S2). Three outlier detection methods identified a total of 719 unique outliers putatively under directional selection (10.9% of all SNPs). The number of outliers identified by each method and analysis, and the overlap between methods, is shown in Figure S2. Using the dataset containing all sampling sites, pcadapt was the least conservative (573 SNPs), OutFLANK was intermediate (134 SNPs), and BAYESCAN was the most conservative (19 SNPs). Seventy‐nine SNPs were identified in at least two outlier methods. Ten SNPs were detected by all three methods, constituting the putatively adaptive dataset. For the inner Bay dataset, pcadapt was the least conservative (633 SNPs), OutFLANK was intermediate (14 SNPs), and BAYESCAN was the most conservative (6 SNPs). Five SNPs were detected by all three methods, constituting the putatively adaptive inner Bay dataset.

The critical R^2^ calculated from the intrachromosomal LD analysis was 0.0989 (root transformed 95th percentile of intrachromosomal LD; Breseghello & Sorrells, 2006). The point at which the loess curve (fit to the intrachromosomal LD) intercepted the critical R^2^ was determined as the average LD decay within each chromosome. Based on these criteria, SNPs were thinned within each chromosome (1 = 250 bp, 2 = 1000 bp, 4 = 330 bp, 6 = 1850 bp; remaining chromosomes (5, 7–10) were not thinned based on this criteria due to loess curve being below critical *R* ^2^ shown in Figure S2). After removing linked SNPs, the thinned neutral dataset consisted of 4641 SNPs.

The critical *R* ^2^ calculated from the intrachromosomal LD analysis for the inner Bay dataset was 0.1026 (Breseghello & Sorrells, 2006). The point at which the loess curve (fit to the intrachromosomal LD) intercepted the critical R^2^ was determined as the average LD decay within each chromosome. Based on these criteria, SNPs were thinned within each chromosome (1 = 180 bp, 2 = 800 bp, 4 = 240 bp; remaining chromosomes (3, 5–10) were not thinned based on these criteria due to loess curve being below critical *R* ^2^). After removing linked SNPs, the thinned neutral dataset consisted of 4922 SNPs.

### 3.2 Genetic diversity and effective population size of natural and restored oysters

To explore patterns of genetic diversity among populations, mean expected heterozygosity (*H*_e_), observed heterozygosity (*H*_o_), allelic richness (A_r_), inbreeding coefficients (*F*_IS_), relatedness, and effective population size (*N*_e_) were calculated for each sampling site using the thinned neutral dataset. *H*_e_ was similar between sampling sites, ranging from 0.218 to 0.239, while *H*_o_ differed more substantially among sites ranging from 0.183 to 0.246 (Table 2). All samples displayed higher levels of *H*_o_ than *H*_e_ except HCS (restored), HCW (MD natural), LC (MD natural), BR, and TS (VA nautral). The Harris Creek restored sample HCS displayed the lowest *H*_o_ overall (0.183), while the restored Harris Creek site HCR1 displayed the highest *H*_o_ overall (0.246). Excluding the single cohort HCS sample, all Harris Creek restored samples displayed slightly higher levels of *H*_o_ than natural Maryland and Virginia populations (Table 2). Allelic richness was similar between sampling sites ranging from 1.932 to 1.98 (Table 2), but showed a strong trend for restored oyster samples, and increased as planting frequency increased (Table 2). The coastal Bay W sample displayed the lowest allelic richness (Table 2). *F*_IS_ values ranged from −0.059 (HCR1) to 0.177 (HCS), and about half of all *F*_IS_ coefficients were negative. The restored mixed‐cohort sites had the lowest *F*_IS_ overall (HCR1, HCR2, HCR4) as well as the coastal Bay (W) site. Global relatedness trends ranged from 0.0022 to 0.0619 (lowest in TS and highest in W; Table 2). Relatedness of mixed‐cohort restored reefs decreased as planting frequency increased and the HCS sample had the lowest relatedness of all restored samples (0.0061). For the Maryland natural samples, LC had the lowest relatedness (0.0056). For the natural Virginia samples, TS had the lowest relatedness and the inner Bay natural Virginia samples had lower relatedness than natural Maryland samples overall (Table 2).

**TABLE 2:** Descriptive statistics for each *C*. *virginica* sampling site, including observed heterozygosity (*H*_o_), expected heterozygosity (*H*_e_), inbreeding coefficient (*F*_IS_) and confidence intervals (CIs), allelic richness (A_r_), effective population size (*N*_e_) and CIs (excluding minor allele frequencies of 0.20 and 0.10), and relatedness Ritland (1996)

Sites	*H*_o_	*H*_e_	*F*_IS_ (CI)	A_r_	*N*_e_ (CI) 0.20	*N*_e_ (CI) 0.10	Ritland
HCR1	0.246	0.233	−0.059 (−0.066, −0.051)	1.963	71.1 (38.5, 244.2)	76.9 (42.1, 257.9)	0.0280
HCR2	0.245	0.234	−0.048 (−0.055 −0.041)	1.971	155.5 (115.5, 232.9)	162.9 (121.8, 241.5)	0.0210
HCR4	0.237	0.230	−0.027 (−0.034, −0.02)	1.978	325.8 (225.3, 574.4)	339.5 (234.9, 598.8)	0.0081
HCS	0.183	0.223	0.177 (0.168, 0.186)	1.972	67.3 (32.9, 310.8)	69.3 (34.1, 316.6)	0.0061
HCW	0.229	0.239	0.043 (0.036, 0.05)	1.980	75.2 (43.3, 188.7)	76.7 (44.2, 192.9)	0.0066
LC	0.225	0.226	0.010 (0.003, 0.017)	1.977	501.4 (362.3, 804.5)	537.7 (386.3, 874.3)	0.0056
TB	0.236	0.231	−0.018 (−0.024, −0.011)	1.972	141.4 (92.1, 278.0)	146.3 (95.5, 286.2)	0.0117
BR	0.220	0.231	0.046 (0.038, 0.052)	1.979	382.8 (188.2, 12461.9)	395.5 (192.8, 29139.3)	0.0038
TS	0.186	0.223	0.166 (0.158, 0.176)	1.975	123.6 (44.5, ∞)	129.4 (47.0, ∞)	0.0022
JR	0.229	0.227	−0.004 (−0.011, 0.004)	1.977	346.3 (216.2, 819.4)	356.0 (221.8, 848.5)	0.0074
W	0.225	0.218	−0.035 (−0.042, −0.027)	1.932	193.3 (118.3, 480.3)	216.1 (131.9, 546.3)	0.0619

Note

HCS *N*_e_ and CIs represent adjusted *N*_e_ and CIs according to Waples et al. (2014). Abbreviations of sampling sites are presented in Table 1.

Estimates of *N*_e_ were variable across sampling sites, ranging from 71.1 (HCR1) to 501.4 (LC) (Table 2). The natural Maryland samples ranged from 75.2 to 501.4, the nautral Virginia samples ranged from 123.6 to 382.8, and the restored Harris Creek samples ranged from 71.1 to 325.8. All but one (TS) of the estimates were bounded at the jackknife confidence limits (jackknife confidence interval range 32.9–12461.9; Table 2). While the adjusted point estimate of *N*_b_ from a single cohort of juveniles was the lowest (67.3), the upper confidence limit was higher than all MD samples except LC and HCR4. The *N*_e_ estimates for the restored Harris Creek samples increased as hatchery‐planting frequency increased and were higher than *N*_e_ estimates from the natural MD populations HCW and TB (Table 2). Overall, the *N*_e_ estimates for the Harris Creek restored samples were similar to the range of values estimated for natural populations in Maryland and Virginia, and confidence limits for the natural and restored populations overlapped substantially.

**FIGURE 2:**
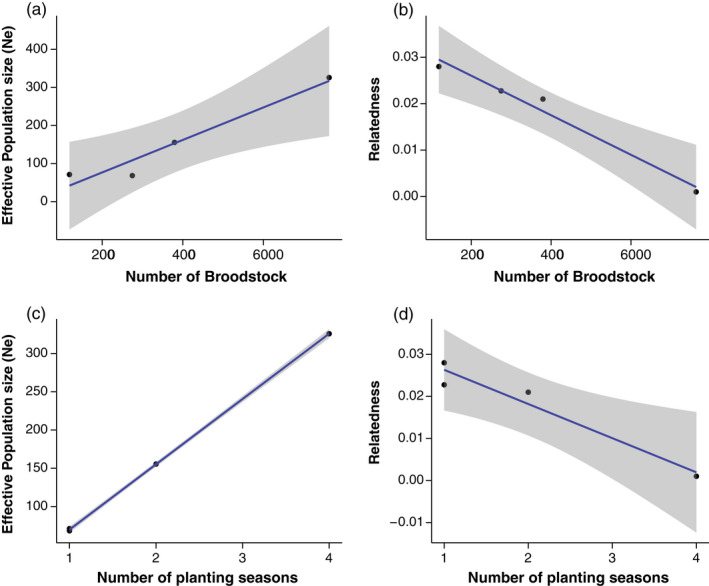
Relationships among genetic diversity of Harris Creek reefs including the number of broodstock and (a) effective population size (*N_e_*) and (b) relatedness and the number of hatchery planting seasons and (c) *N*_e_ and (d) relatedness of restored sites

The number of broodstock used for hatchery plantings was significantly positively associated with *N*_e_ (*p* = 0.030, *R* ^2^ = 0.913; Figure 2a) and significantly negatively correlated with relatedness of restored reefs (*p* = 0.012, *R* ^2^ = 0.964; Figure 2b). The number of hatchery‐planting seasons was significantly positively associated with *N*_e_ (*p* < 0.001, *R* ^2^ = 0.999; Figure 2c) and significantly negatively associated with relatedness of restored reefs (*p* = 0.029, *R* ^2^ = 0.914; Figure 2d). There was a nonsignificant (*p* = 0.11) positive association between the average broodstock sex ratio and *H*_o_ (*R* ^2^ = 0.6939; Figure S3). The model containing the average broodstock sex ratio and the number of planting seasons was somewhat predictive of *H*_o_, but was not statistically significant (*p* = 0.18). Overall, the relationships between genetic diversity metrics and hatchery practices (planting effort and broodstock size) were positive, strong, and highly predictive.

### 3.3 Genetic differentiation, population structure, and population assignment

**FIGURE 3:**
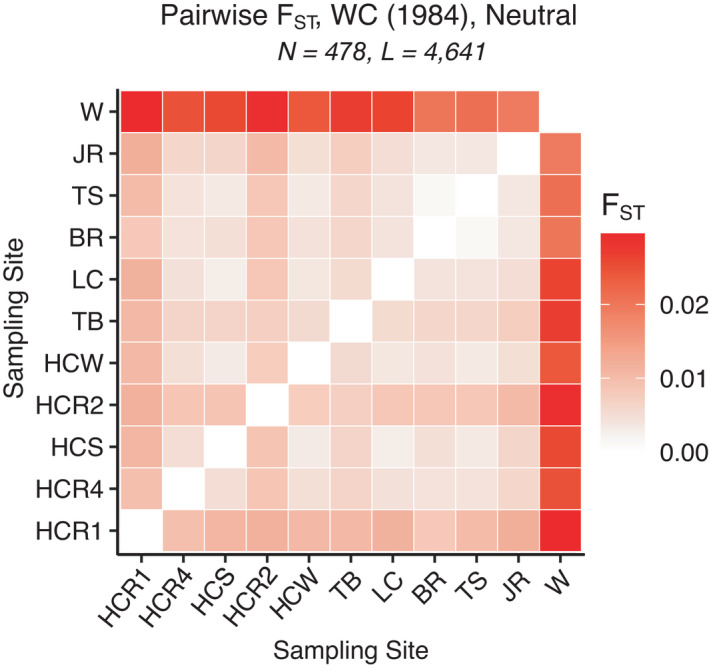
Heatmap of pairwise *F*_ST_ for *C. virginica* populations using the putatively neutral SNPs. Inner Bay populations (HCR1‐JR) are ordered from north to south. Abbreviations of sampling sites are presented in Table 1

**FIGURE 4:**
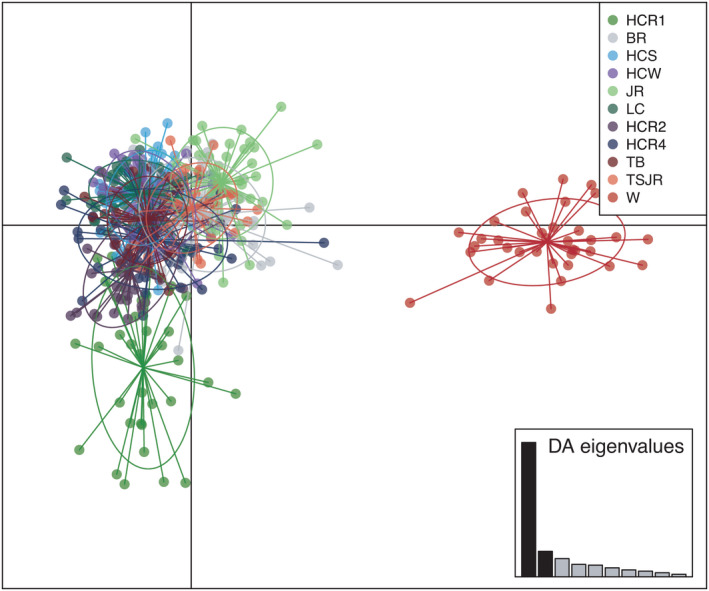
Discriminant analysis of principal components (DAPC) among natural and restored *C. virginica* populations based on 4461 neutral unlinked SNPs. Abbreviations of sampling sites are presented in Table 1

Pairwise *F*_ST_ estimates between natural and restored populations were small, ranging from 0.001 to 0.032 (Figure 3). All pairwise *F*_ST_ estimates were highest between the coastal Bay Wachapreague (W) site and all other sites (0.019 < *F*_ST_ < 0.030). Pairwise *F*_ST_ estimates between HCR1 and the inner Bay sites were higher than comparisons among other inner Bay populations (0.002 < *F*_ST_ < 0.012). Similar to *F*_ST_ results, analyses of population structure via DAPC (49 PCs retained) revealed four major population clusters, with the coastal Bay (W) sample grouping distinctly from all natural and restored inner Bay sites (Figure 4). In addition, subtle genetic differences were observed between the HCR1 site and the rest of the sites from Harris Creek (Figure 4). Analysis in STRUCTURE (Figure S4) also suggested four clusters based on both the mean likelihood values (L(K)) and the Evanno method (deltaK). Finally, Mantel tests showed a significant correlation between pairwise *F*_ST_ and water distance for the neutral dataset, indicating a moderate trend of isolation by distance (adjusted *R* ^2^ = 0.257, *p* = 0.001; Figure S5a), even when restored samples were removed (*R* ^2^ = 0.1977, *p* = 0.014; Figure S5b).

### 3.4 Genotype–environment associations

**FIGURE 5:**
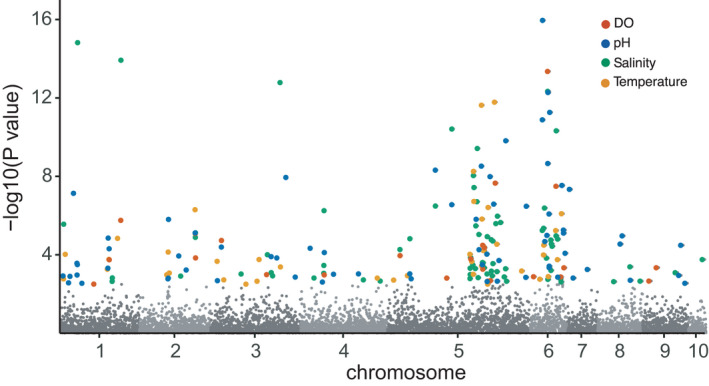
Manhattan plot showing *p*‐values from RDA for 6654 SNPs aligned by position on chromosomes 1–10. Colored dots correspond to 208 SNPs identified as outliers by RDA that were correlated with environmental parameters (salinity, temperature, DO, and pH). Note significant clustering of RDA outliers on chromosomes five and six

To identify genes along environmental gradients that are indicative of local adaptation, allele frequencies were examined for association with environmental variables using the multivariate RDA approach. Using RDA on the full filtered dataset (6654 SNPs; on 2 retained axes), a total of 208 SNPs were significantly associated with the five environmental variables tested, with a large proportion of SNPs being more specifically attributed to mean salinity (74 SNPs), pH variables (69 SNPs total; 39 SNPs mean pH and 30 SNPs min pH), minimum water temperature (41 SNPs), and minimum dissolved oxygen (DO; 24 SNPs). Interestingly, most of the SNPs associated with environmental variables were located on chromosomes one through six. However, many of the SNPs associated with environmental variables were located across all ten chromosomes (Figure 5). Using RDA on the inner Bay dataset (6654 SNPs), 48 SNPs were significantly associated with the five environmental variables tested, with a large proportion of SNPs being more specifically attributed to minimum salinity (19 SNPs), maximum water temperature (10 SNPs), DO variables (10 SNPs total; 5 SNPs mean DO and 5 SNPs min DO), and minimum pH (9 SNPs). Information on SNPs correlated with the environmental predictors is presented in Table S3.

### 3.5 Effect of environmental variables and geography on genetic variation

For the RDA using the neutral, unlinked datasets (full and inner Bay‐only sites), two geographic variables using principal coordinates of neighbor matrices (PCNM1 and PCNM2) and four environmental variables (mean salinity, mean water temperature, minimum DO, and mean pH) were selected for RDA. The RDA explained a small but significant portion of the genetic variation for all sites (*R* ^2^_adj_ = 1.1%, *p* = 0.001; Table 3) as well as the inner Bay sites (*R* ^2^_adj_ = 0.65%, *p* = 0.001; Table 3). Partitioning of total variance analysis indicated that the environment had a greater unique contribution to genetic variation compared with geography (65.5% vs. 27.8% for all sites and 65.3% vs. 31.2% for inner Bay sites). The proportion of genetic variation explained by the environment that was also spatially structured was similar for both the full (all populations) dataset and the inner Bay‐only dataset (6.7% and 3.5%, respectively). For the full dataset, mean salinity, PCNM1, and mean pH were the most important predictors of neutral genetic variation among all variables considered, respectively. For the inner Bay dataset, PCNM1, mean water temperature, and mean salinity were the most important predictors of neutral genetic variation among all variables considered, respectively (Table 4).

**TABLE 3:** Redundancy analysis (RDA) results for neutral and adaptive SNP datasets including all sites and only the inner Bay sites (excluding Wachapreague). The environmental parameters include mean salinity (MS), mean water temperature (mWT), mean pH (mpH), and minimum dissolved oxygen (DO; minDO). Environmental variables are ordered according to significance in RDA

Dataset	SNP genetic variation	Partitioned variance	Proportion constrained	Adjusted *R* ^2^	*p*‐value
*All sites*					
4641 neutral SNPS	Total variance	1089			
	Full model: Spatial + Environment (constrained variance)	25.3	0.0234	0.011	0.001
	Environment (MS + mWT + minDO + mpH) | Spatial	16.6	0.008012	0.007	0.001
	Spatial (PCNM1 + PCNM2) | Environment	7.03	0.006453	0.0023	0.001
	Spatial ∩ Climate	1.67	0.008935	0.0017	NA
Ten SNPS adaptive SNPs	Total variance	4.658			
	Full model: Spatial + Environment (constrained variance)	1.067	0.229	0.219	0.001
	Environment (MS + mWT + minDO + mpH) | Spatial	0.7432	0.156	0.154	0.001
	Spatial (PCNM1 + PCNM2) | Environment	0.0752	0.21289	0.013	0.001
	Spatial ∩ Climate	0.25	0.062	0.052	NA
*Inner Bay sites*					
4922 neutral SNPS	Total variance	1136			
	Full model: Spatial +Environment	22.65	0.01994	0.0065	0.001
	Environment (MS + mWT + minDO + mpH) | Spatial	14.79	0.013	0.004	0.001
	Spatial (PCNM1 + PCNM2) | Environment	7.06	0.0062	0.0017	0.001
	Spatial ∩ Climate	0.8	0.00074	0.0008	NA
Five adaptive SNPs	Total variance	2.33055			
	Full model: Spatial + Environment (constrained variance)	0.22853	0.0981	0.0856	0.001
	Environment (MS + mWT + MinDO + mpH) | Spatial	0.18391	0.07894	0.071	0.001
	Spatial (PCNM1 + PCNM2) | Environment	0.03743	0.01606	0.012	0.001
	Spatial ∩ Climate	0.00719	0.0031	0.0026	NA

Note

Significance of the global model and significance of each variable in the partial RDA were evaluated using an ANOVA (10,000 permutations).

**TABLE 4:** Results of redundancy analyses (RDA) on genetic variation. Significance of individual significant environmental variables in RDAs. Variables shown were all significantly associated with genetic variation

Dataset	Significant variable	Variance	*F*	*p*‐value
*All sites*				
4641 neutral SNPS	MS	5.4	2.3898	0.001
	mWT	3.74	1.6566	0.001
	MinDO	3.61	1.5999	0.001
	mpH	3.83	1.6962	0.001
	PCNM1	5.35	2.3709	0.001
	PCNM2	3.37	1.4924	0.001
Ten SNPS adaptive SNPs	MS	0.4631	60.7373	0.001
	mWT	0.1769	23.1952	0.001
	MinDO	0.0646	8.4692	0.001
	mpH	0.0386	5.0686	0.002
	PCNM1	0.2649	34.7384	0.001
	PCNM2	0.0588	7.7156	0.001
*Inner Bay sites*				
4922 neutral SNPS	MS	3.75	1.4679	0.001
	mWT	4.03	1.5793	0.001
	MinDO	3.58	1.4019	0.001
	mpH	3.43	1.3434	0.001
	PCNM1	4.37	1.712	0.001
	PCNM2	3.59	1.3667	0.001
Five adaptive SNPs	MS	0.02162	4.4847	0.005
	mWT	0.10982	22.7796	0.001
	MinDO	0.038	7.8829	0.001
	mpH	0.01453	3.0147	0.023
	PCNM1	0.02945	6.1089	0.002
	PCNM2	0.01509	3.1307	0.023

For the RDA using SNPs identified as being putatively adaptive for the full dataset (10 SNPs) and for the inner Bay sites (5 SNPs), two geographic (PCNM1 and PCNM2) and four environmental variables (mean salinity, mean water temperature, minimum DO, and mean pH) were selected for RDA. The RDA explained a moderate but significant portion of the genetic variation for all sites (*R* ^2^_adj_ = 21.9%, *p* = 0.001; Table 3) and a small but significant portion of genetic variation for the inner Bay sites (*R* ^2^_adj_ = 8.56%, *p* = 0.001; Table 3). Partitioning of total variance analysis indicated that the environment had a greater unique contribution to genetic variation compared with geography (69.7% vs. 7% for all sites and 80.5% vs. 16.4% for inner Bay sites). The proportion of genetic variation explained by the environment that was also spatially structured was slightly different between datasets (23.3% all sites vs. 3.1% for inner Bay sites). For the full dataset, mean salinity, PCNM1, and mean water temperature were the most important predictors of adaptive genetic variation among all variables considered, respectively (Table 4). For the inner Bay dataset, mean water temperature, PCNM1, and mean salinity were the most important predictors of adaptive genetic variation among all variables considered, respectively (Table 4).

### 3.6 Functional annotation of outliers

The SNPs identified as outliers in at least two genome‐scan methods and in RDA from both datasets (208 SNPs) were distributed across all 10 chromosomes. Of all the SNP‐containing sequences that were BLASTed against the protein sequences of the eastern oyster genome, 128 SNPs had significant hits (minimum *e*‐value of 0.001), 85 of which had gene ontology (GO) annotations, while 35 were uncharacterized proteins. For the inner Bay dataset, SNPs identified in at least two genome‐scan methods and in RDA (90 SNPs) were distributed across all 10 chromosomes, 68 had significant hits, 47 of which had gene ontology (GO) annotations, while 18 were uncharacterized proteins. For the full dataset and inner Bay dataset, most of the genes were involved in ion binding and transmembrane transporter activity. A complete list of significant GO terms and candidate genes for the full dataset is in Table S4.

## 4 DISCUSSION

Assessing patterns of neutral and adaptive genetic variation is critical to establishing restoration programs that aim to preserve natural or current patterns of genetic diversity and genetic structure, and promote resilience in the face of environmental change. However, for marine bivalve species with complex life‐history features, this information is often unavailable or is rarely integrated into restoration strategies. A RADseq genotyping approach was used to characterize patterns of genetic variation within and among natural and restored eastern oyster populations in the Chesapeake Bay, and the high‐resolution data were used to investigate population structure, local adaptation, and the extent at which environmental gradients influence genetic variation among these populations.

There are four major findings of this study that provide critical information for management and restoration of eastern oysters, which typify the periodic, broadcast‐spawning life history of many other marine animal species (e.g., Winemiller & Rose, 1992). First, restored oyster reefs in Harris Creek, MD, had similar levels of genetic diversity compared with proximal natural populations. Second, the number of broodstock used for spat production and the frequency of restoration planting had strong, positive associations with metrics of genetic diversity including *N*_e_ and relatedness. Third, despite previous restoration efforts, frequent historical translocations, and high dispersal potential of oyster larvae, we uncovered a moderate degree of neutral population genetic structure in natural and restored Chesapeake Bay oyster populations, which is consistent with previous studies (e.g., Rose et al., 2006; Turley et al., 2019) and suggests that fine‐scale population structure can exist over small scales for marine bivalves. Finally, strong correlations between environmental variables and outlier SNPs were found, which suggests that local adaptation or genotype‐by‐environment interactions may be driving the adaptive differentiation of oysters over relatively small scales. Overall, these results add to the growing evidence for fine‐scale genetic structure and potential for local adaptation in marine animal species. Moreover, these results suggest that sourcing natural broodstock from large, local populations experiencing similar environments to candidate sites is likely to be most appropriate for hatchery‐based restoration of oysters.

### 4.1 Comparison of genetic diversity and *N*_e_ between restored vs. natural oysters

In general, estimates of genetic diversity in the Chesapeake Bay oyster populations sampled were comparable to those in previously published studies. Notably, restored oysters from Harris Creek had comparable levels of genetic diversity to natural oysters from Maryland and Virginia. More than half (6/11) of the estimated inbreeding values (*F*_IS_) across sampling locations were negative, indicating heterozygosity excess, and those that were positive were lower than those observed using SNP datasets in Canadian eastern oyster populations (*F*_IS_ = 0.191–0.211; Bernatchez et al., 2019), and other oyster species, such as the black lip pearl oyster (*F*_IS_ ≥ 0.5; Lal et al., 2018), but similar to those of the Sydney rock oyster (*F*_IS_ = 0.1465–0.2093; O’Hare et al., 2021). Inbreeding levels were also lower than what was observed in a recent study of oyster populations in the lower Chesapeake Bay using 48 SNPs (*F*_IS_ = 0.02–0.156, Turley et al., 2019), and in Rhode Island using microsatellites (*F*_IS_ = 0.00–0.47; Jaris et al., 2019), and comparable to those observed in a recent study of the Olympia oyster using genome‐wide SNPs (*F*_IS_ = –0.09–0.133; Silliman, 2019). Mean heterozygosity (*H*_o_ and *H*_e_) was within the range of or slightly lower than what has been observed in studies using similar markers (SNPs) in oysters. For example, observed heterozygosity levels were similar to those observed in Canadian eastern oyster populations (Bernatchez et al., 2019), but lower than what was observed in Delaware Bay oysters (0.329–0.343; Thongda et al., 2018) and higher than what was observed in the Sydney rock oyster (0.1207–0.1367; O’Hare et al., 2021). Relatedness of restored and natural populations was similar to values previously reported in natural (0.002–0.011) and restored (0.012) populations in the Chesapeake Bay (Hornick & Plough, 2019) and substantially lower than that of hatchery‐produced offspring (0.03–0.129). Overall, these results suggest that genetic diversity of restored and natural oyster populations in the upper Chesapeake Bay are comparable and that large‐scale hatchery‐based restoration has not caused significant declines in diversity, at least based on the reefs sampled and metrics examined. This was found previously (Hornick & Plough, 2019), albeit with limited sampling and marker resolution.

In general, *N*_e_ estimates were similar in the magnitude of values reported for eastern oyster populations in other regions of the US east coast. For example, estimates of *N*_e_ were similar to previous estimates for oysters in the Delaware Bay (37–437; He et al., 2012), in the James River (535–1 516; Rose et al., 2006), and in the Choptank River (68.3–178.2; Hornick & Plough, 2019), but are higher than those reported in the Delaware Bay (33.8) by Hedgecock et al. (1992). However, Chesapeake Bay *N*_e_ estimates, these and others, are much lower than those we estimated using data from Bernatchez et al. (2019) for Canadian oyster populations, which utilized a similar RADseq genotyping approach (examined genome‐wide SNPs; Table S5). Point estimates of *N*_e_ from this study (and associated confidence intervals) are consistently an order of magnitude lower than *N*_e_ estimates from Canadian populations (*N*_e_ range 236.8–7071.7, Table S5), except for one Canadian population (COC), which was of a similar order of magnitude to the Chesapeake Bay population estimates (Table S5). The difference between US versus Canadian population estimates may be due to a number of environmental, exploitative, and demographic differences between the regions, and we acknowledge the caveats associated with comparing these two RADseq datasets (e.g., different restriction enzymes used and different numbers of SNPs examined), as well as the numerous caveats associated with *N*_e_ estimation (Waples et al., 2013, 2014, 2016). Still, the differences are substantial, and it is possible that the *N*_e_ of Canadian populations is much larger than populations along the US east coast, which have experienced more intensive harvest pressure and human impacts leading to population declines (Beck et al., 2011). Based on the comparison of current vs. historical abundances of native oyster reefs, the condition of oyster reefs in Canada is characterized as “fair” (50% to 89% lost) compared to the characterization for Chesapeake Bay oyster reefs, which is “poor” (90% to 99% lost; Beck et al., 2011). The finding that restored reefs in the Chesapeake Bay exhibit similar genetic diversity to natural populations in the region is important, but perhaps less impressive if one considers that substantial population declines of oysters have occurred in the Chesapeake Bay over the last century (Beck et al., 2011; Rothschild et al., 1994; Wilberg et al., 2011). Thus, comparisons between restored and contemporary natural reefs overlook the potentially large differences between present and historical diversity (i.e., shifting baselines; Pauly, 1995). If these estimates of *N*_e_ in Canadian populations are accurate, and if they are broadly reflective of reduced anthropogenic impacts over time (e.g., lower fishing pressure; Beck et al., 2011), *N*_e_ of Chesapeake Bay oyster populations (natural or restored) are still much reduced compared to what they likely were in the past. Therefore, maintaining diversity of extant Chesapeake Bay natural populations should only be a minimum target.

### 4.2 Effect of planting frequency and broodstock size on restored reef genetic diversity

The number of broodstock used for hatchery plantings and the number of hatchery‐planting seasons significantly impacted diversity at restored reefs in Harris Creek. We found significant and strongly predictive positive relationships between planting effort and broodstock size and genetic diversity metrics (*N*_e_ and relatedness), as well as a positive correlation between broodstock male‐to‐female ratio and observed heterozygosity. To date, few studies have assessed how hatchery production techniques can directly (and positively) impact genetic diversity of cultured and supplemented populations of bivalves. A somewhat similar result was found in a recent study of eastern oysters, in which the ratio of males‐to‐females in broodstock was positively correlated with metrics of genetic diversity of hatchery‐produced eastern oyster cohorts (Hughes et al., 2019). However, Hughes et al. (2019) did not focus on restoration specifically and the experiments conducted were on a much smaller scale. Using individual‐based model simulations, Katalinas et al. (2019) investigated how stock enhancement practices, such as the number of breeders and relative contribution of stocked fish, impact levels of genetic diversity on the wild spawning population of red drum in South Carolina (Katalinas et al., 2019). Model results indicated that in order to maintain genetic diversity of the wild population, the stock enhancement program should use at least 10 effective breeders in the hatchery (replaced annually), with mean contributions of stocked fish at less than 30% (Katalinas et al., 2019). Future simulation‐based work incorporating bivalve life‐history features and empirical genetic data would be useful for quantifying genetic diversity changes associated with varying hatchery practices (Hornick, 2020). It is clear that the use of large numbers of broodstock numbers from multiple local sites and the planting of multiple cohorts over many planting seasons can increase diversity of restored sites, especially when initial broodstock numbers are limited. More empirical work is needed to understand how hatchery practices directly influence genetic diversity of supplemented populations, especially in species with complex life‐history features that may make maintaining genetic diversity in the hatchery more challenging (e.g., Hornick & Plough, 2019). Nevertheless, these modifiable hatchery or husbandry practices (broodstock number, male‐to‐female ratio of broodstock, and planting frequency) may offer a straightforward way to achieve short‐term goals of increasing abundances while also approaching long‐term goals of maintaining diversity and promoting self‐sustaining natural populations.

### 4.3 Fine‐scale population structure and adaptive divergence

Contemporary population structure of Chesapeake Bay eastern oysters results from diverse factors including larval dispersal and behavior, natural selection over environmental gradients, genetic drift, and demographic history. Though weak or negligible genetic structure is often assumed for marine broadcast‐spawning species over small spatial scales (e.g., 10s of km; Bradbury et al., 2008), subtle but significant population structure among oyster populations in the Chesapeake region was uncovered, which was evidenced by genetic clustering of proximal sites and significant isolation by distance (IBD) over the length of the estuary. Previous work using eight microsatellite markers (Rose et al., 2006) and 41 SNPs (Turley et al., 2019) resolved significant genetic differences within Chesapeake Bay oyster populations and demonstrated a subtle pattern of IBD on spatial scales similar to the geographic scale encompassed by our study. However, these results contrast with the expectation that decades of replenishment and restoration activities in Maryland and Virginia, which resulted in substantial movement of oysters (Kennedy et al., 2011; Schulte, 2017), would homogenize allele frequencies and limit any signatures of environmental and geographic population structure. Given the results reported here and the fact that larval periods of 2–3 weeks should allow for dispersal distances well beyond the scale of genetic structure found, it seems likely that the heterogeneous estuarine environment is driving at least some of the observed genetic structure. RDA indicated that environmental gradients had a stronger effect on genetic variation than distance‐based isolating factors, such as genetic drift. Environmental factors were also found to play a critical role in the distribution of neutral and putatively adaptive genetic variation in oysters from the Maritime Provinces in Canada, which have experienced less fishing pressure and human‐assisted migration (Bernatchez et al., 2019). Alternatively, it is possible that the observed patterns of genetic structure may, in part, reflect ancestral population structure present before widespread movement of oysters that occurred over the last 150 years (Kennedy & Breisch, 1983; Kennedy et al., 2011), though more work would be required to test this hypothesis. Future studies incorporating coalescent modeling approaches could provide important information regarding the historical relationships of Chesapeake Bay oyster populations (e.g., Chen et al., 2017; Díaz et al., 2018).

The RDA indicated that salinity was the most important predictor of both neutral and adaptive variation. The RDA approach has been used to study the influence of the environment on genetic structure of commercially important bivalves such as the eastern oyster (*Crassostrea virginica*; Bernatchez et al., 2019), the Atlantic deep‐sea scallop (*Placopecten magellanicus*; Lehnert et al., 2019), pearl oyster (*Pinctada fucata*; Takeuchi et al., 2020), and the common cockle (*Cerastoderma edule*; Coscia et al., 2020). The observed neutral population structure uncovered in this study may be related to the influence of salinity on larval dispersal. Salinity influences larval duration, growth, and survival (Davis, 1958; Hidu & Haskin, 1978; Kennedy, 1996; Scharping et al., 2019) during the period when oysters disperse. Salinity is also a critical factor that cues vertical swimming behavior and transport of oyster larvae in laboratory (Hidu & Haskin, 1978; Newell et al., 2005), field (Carriker, 1951; Nelson & Perkins, 1931), and modeling studies (Dekshenieks et al., 1996; Narváez et al., 2012). Recent studies have demonstrated that survival of eastern oyster larvae is influenced by the salinity at gametogenesis and the genetic background of broodstock (e.g., Eierman & Hare, 2014; Newkirk, 1978; Scharping et al., 2019) and that salinity tolerance in oysters is genetically based (Griffiths et al., 2021; McCarty et al., 2020). While oysters lack the ability to adjust extracellular fluids, they have compensatory machinery for transporting osmotically active solutes including free amino acids (FAAs) (Pierce & Amende, 1981; Zhao et al., 2012). Genes correlated with salinity in our RDA analyses were, as expected, involved in osmoconformation, hydrolase activity, and metabolism (Table S4). Previous studies have demonstrated the direct link between response to osmotic stress and hydrolase activity (Jones et al., 2019) and phosphorylation (Eierman & Hare, 2014) in eastern oysters. Additional studies have demonstrated the link between response to osmotic stress and increased oxygen uptake in the Pacific oyster (Pack et al., 2021) and the blue mussel (Hawkins & Hilbish, 1992). Many of the SNPs associated with salinity were located on chromosomes five and six (Figure 5). However, significant SNPs associated with salinity and other environmental variables were also located across chromosomes one through six, which is suggestive of locally adapted variation being pervasive throughout multiple genomic regions (i.e., is polygenic). In addition, these results demonstrate the complexity of selection patterns and that allelic variation depends on more than one environmental gradient. We recognize that the range of values for other environmental variables were not as dynamic as those of salinity in this study (Table S6), and the sampling resolution of the environmental data was limited (twice each month). Future studies that include finer resolution within‐bay population and environmental sampling may reveal additional patterns of selection and differentiation, which could impact the broad‐scale correlations observed here. Nevertheless, these results provide insight into the mechanisms of salinity adaptation in oysters, and how other important environmental variables (e.g., DO, pH, and temperature) drive patterns of genetic variation over small spatial scales.

### 4.4 Implications for restoration

Results from this study provide evidence that oyster populations in the Chesapeake Bay may be locally adapted to prominent environmental features, particularly salinity, which has direct management implications. First, the finding of local adaptation over small spatial scales suggests limiting introgression from divergent populations (Conover, 1998; do Prado et al., 2018) by favoring the use of local natural broodstock for restoration. Collecting local broodstock could be beneficial because nearby populations are likely to be more connected by gene flow and experience similar environments. However, because geographic distance did not significantly predict neutral or adaptive variation, matching environmental conditions of collection and restoration sites may be more important for broodstock and/or seed selection than geographic distance alone (Bischoff & Hurault, 2013; McKay et al., 2005). This result is particularly important for restoration of estuarine species, as stark environmental change over small geographic scales is common (e.g., Elliott & McLusky, 2002; McLusky & Elliott, 2007). Whether the use of local broodstock actually results in increased survival of planted individuals merits future investigation, as results of previous studies have been mixed. For example, Bible and Sanford (2016) performed reciprocal transplants of Olympia oyster offspring from three sites in San Francisco Bay and found that oysters of local origin tended to survive better than locally nonadapted sources. These results suggested that local adaptation may occur even within a single estuary (Bible & Sanford, 2016). A recent experimental study of larval eastern oysters showed that survival at a given salinity seems to be matched to the salinity of the parental population (or conditioning salinity; Scharping et al., 2019). Whether this survival is a result of adult acclimation vs. local adaptation will require future work. However, local sources do not always perform better than all other sources (Hereford, 2009; Leimu & Fischer, 2008). A recent study of eastern oysters documented significant genetic by environment variation in survival and growth, but no evidence for local adaptation (Hughes et al., 2017). Thus, the benefits of using local broodstock may be subtle, and likely depend on idiosyncrasies of the specific set of populations under study, including the amount of standing diversity in the system, genetic background, and the connectivity among populations. Restoration plans aimed at conserving multiple, interconnected reefs will likely capture an important axis of adaptive variation and maintain genetic diversity of restored populations. Future work should incorporate a larger number of populations to quantify the spatial scale of local adaptation (Hice et al., 2012) and include reciprocal transplant experiments to determine if local populations perform better than non‐local counterparts.

Despite the potential benefits of using locally‐adapted broodstock, broodstock collection from local populations may not be ideal or feasible for restoration in some cases. In areas such as Australia and Europe, native populations of bivalves have been driven to local extinction, so sourcing broodstock locally is typically not an option (Beck et al., 2011). In addition, some local oyster populations may lack a sufficient amount of genetic variability to adapt to rapidly changing climatic conditions (Harris et al., 2006; Jones, 2013; McKay et al., 2005; Montalvo et al., 1997; Rice & Emery, 2003), or they may have small *N*_e_ and high levels of inbreeding (Leimu et al., 2006). In such cases, sourcing broodstock from a number of local and/or regional populations may be the only solution. As shown in this study, environmental gradients and the rate of gene flow can vary, so it remains difficult to prescribe a standard geographic distance as a scale for local adaptation. In the Chesapeake Bay, the availability of fine‐scale environmental data can potentially aid in identifying the drivers of adaptive differences between reefs rather than just identifying the effects of geographic distance, which could allow delineation of zones by environmental distance and possibly guide broodstock selection. The idea of using a more widely available “coarsely adapted” mixture of broodstock sources that provide a reservoir of genetic variation for natural selection to act on is attractive and may increase restoration success by increasing species’ adaptive potential (Lesica & Allendorf, 1999; Rice & Emery, 2003). Nevertheless, these results indicate that when considering broodstock sources based on adaptive differentiation in heterogeneous environments, collecting broodstock from large populations with similar environments to candidate sites may increase population sustainability.

## 5 CONCLUSIONS

This study provides comprehensive characterization of neutral and adaptive population structure of restored and natural oysters in the Chesapeake Bay and is the first study to investigate genetic changes of restored reefs associated with variable hatchery‐planting frequencies. The results obtained here suggest that using large numbers of local, natural broodstock in hatchery‐based restoration programs and planting of reefs multiple times (especially if broodstock numbers are low) can increase diversity. Furthermore, results from this study contribute to the growing body of evidence that adaptive differentiation can occur over very fine geographic scales in marine species and suggest that this structuring is at least partly driven by spatial heterogeneity in environmental parameters such as salinity, temperature, pH, and dissolved oxygen. The eastern oyster is a commercially exploited species with large‐scale restoration efforts underway in the Chesapeake Bay and in other regions (e.g., Brumbaugh & Coen, 2009; Dinnel et al., 2009; Holley et al., 2018). An understanding of spatial patterns of neutral and adaptive genetic differentiation can inform restoration strategies and potentially increase the sustainability of restored oyster populations in the future.

## CONFLICT OF INTEREST

None declared.

## Supporting information

Table S1–S6

Supplementary Material

## Data Availability

Data for this study will be available at the Dryad Digital Repository https://datadryad.org/stash/dataset/doi:10.5061/dryad.47d7wm3fm. [Correction added on 12 Jan 2022, after first online publication: The Dryad link has been included in this version]
