# Supplementary material for: Genome‐wide analysis of natural and restored eastern oyster populations reveals local adaptation and positive impacts of planting frequency and broodstock number
Source: Evol Appl. 2021 Dec 7;15(1):40–59. doi: 10.1111/eva.13322 (PMC8792482; doi:10.1111/eva.13322)
Supplement: Supplementary file 1 — Table S1–S6 [file EVA-15-40-s002.docx]

**Supplementary Tables S1-S6.**

**Table S1.** Number of broodstock and broodstock sex ratio (1-male:female), size (acres) of restored sites, and number of planting seasons for each of the restored sites for *C. virginica* in Harris Creek from this study (HCR1, 2, and 4) as well as the restored site from Hornick and Plough (2019) (HCR1-2).

| **Site** | **Plantings** | **Size (acres)** | **Broodstock** | **Broodstock sex ratio** |
| --- | --- | --- | --- | --- |
| HCR1 | 1 | 7.72 | 1189 | 0.632 |
| HCR2 | 2 | 3.65 | 3795 | 0.675 |
| HCR4 | 4 | 4.62 | 7631 | 0.679 |
| HCR1-2 | 1 | 6.56 | 2748 | 0.573 |

Abbreviations of Chesapeake Bay sampling sites are presented in Table 1.

**Table S2.**  Summary of data filtering procedures: rows refer to filtering steps; columns refer to statistics for each step. For columns, ‘sites’ refers to individual polymorphisms (SNPs, indels, or complex polymorphisms), and ‘Inds’ refers to individuals. ‘Start’, ‘End’, and ‘Removed’ refer, respectively, to the number of each unit before the filtering step, the number after the filtering step, and the number removed with the filter.

| **Filter** | **Start sites** | **End sites** | **Start Inds** | **End Inds** | **Removed sites** | **Removed Inds** |
| --- | --- | --- | --- | --- | --- | --- |
| Minor allele count <3, Mean site quality < 20, & Mean site call rate < 0.5 | 4544551 | 538478 | 570 | 570 | 4006073 | 0 |
| Minimum depth =5 | 538478 | 538478 | 570 | 570 | 0 | 0 |
| Filter_missing_ind script; Ind call rate < 0.5 | 538478 | 538478 | 570 | 491 | 0 | 79 |
| Minor allele frequency < 0.05, mean site call rate < 0.9 | 538478 | 15731 | 491 | 491 | 522747 | 0 |
| pop_missing_filter script; call rate 0.75 in one population | 15731 | 14725 | 491 | 491 | 1006 | 0 |
| dDocent_filters script | 14725 | 7040 | 491 | 491 | 7685 | 0 |
| Decomposed to allelic primitives | 8163 | 7832 | 491 | 491 | 331 | 0 |
| Hardy-Weinberg equilibrium | 7832 | 7796 | 491 | 491 | 36 | 0 |
| Max alleles=2 | 7796 | 7710 | 491 | 491 | 86 | 0 |
| Remove reps | 7710 | 7710 | 491 | 478 | 0 | 13 |

**Table S3.** Redundancy analysis (RDA) results for the full dataset including all populations (6,654 SNPs) and the inner Bay populations (excluding Wachapreague; 6,654 SNPs). Correlated represents SNPs identified as outliers in RDA and correlated with environmental variables.

| **Environmental Variable** | **All populations** | | **Inner Bay populations** | |
| --- | --- | --- | --- | --- |
| Mean salinity | 74 | NA | |  |
| Minimum salinity | NA | 19 | |  |
| Minimum water temp | 41 | NA | |  |
| Maximum water temp | NA | 10 | |  |
| Minimum pH | 30 | 9 | |  |
| Mean pH | 39 | NA | |  |
| Minimum DO | 24 | 5 | |  |
| Mean DO | NA | 5 | |  |

**Table S4.** Significant BLAST matches from sequences identified as being putatively under selection from oyster *Crassostrea virginica* populations in the studied region (full dataset 6,654 SNPs and all populations; 128 SNPs total). SNPs are located in the eastern oyster genome and chromosomes. Protein IDs and names are derived from the eastern oyster protein sequences of the genome. Gene ontologies related to the identified protein (GO ID) were retrieved using protein names and the environmental variable correlated with proteins is listed (MinDO: minimum dissolved oxygen, MS: mean salinity, MinWT: minimum water temperature, MpH: mean pH, and MinpH: minimum pH).

| **Correlated variable** | **Chromosome** | **SNP Position** | **Protein ID** | **Protein name** | **E-value** | **GO ID** | **Enzyme Code** | **Enzyme Name** |
| --- | --- | --- | --- | --- | --- | --- | --- | --- |
| MinDO | chr1 | 54741363 | XP_022303022.1 | uncharacterized protein LOC111110719 | 2 x 10^-6^ |  |  |  |
| MinDO | chr5 | 68226186 | XP_022342720.1 | tripartite motif-containing protein 5-like | 1 x 10^-16^ | GO:0043167 |  |  |
| MinDO | chr5 | 69811720 | XP_022338681.1 | uncharacterized protein LOC111134143 isoform X1 | 5 x 10^-15^ | GO:0043167 |  |  |
| MinDO | chr5 | 70332957 | XP_022340940.1 | circadian locomoter output cycles protein kaput-like | 3 x 10^-99^ | GO:0003674;GO:0009058;GO:0034641 |  |  |
| MinDO | chr5 | 72463351 | XP_022291782.1 | uncharacterized protein LOC111103071 isoform X2 | 2 x 10^-27^ |  |  |  |
| MinDO | chr6 | 8303728 | XP_022288266.1 | arachidonate 15-lipoxygenase B-like isoform X2 | 3 x 10^-37^ | GO:0016491;GO:0043167 | EC:1.13.11 | Acting on single donors with incorporation of molecular oxygen (oxygenases). The oxygen incorporated need not be derived from O(2) |
| MinDO | chr6 | 42645074 | XP_022286415.1 | AT-rich interactive domain-containing protein 5B-like isoform X3 | 3 x 10^-99^ | GO:0003677;GO:0005634;GO:0009058;GO:0034641 |  |  |
| MinDO | chr6 | 32371906 | XP_022286837.1 | maltase-glucoamylase%2C intestinal-like | 5 x 10^-21^ | GO:0005975;GO:0016798 |  |  |
| MinDO | chr9 | 53281020 | XP_022305110.1 | uncharacterized protein LOC111112093 | 1 x 10^-73^ |  |  |  |
| MinDO | chr9 | 19046082 | XP_022305196.1 | mucin-2-like isoform X8 | 2 x 10^-27^ |  |  |  |
| MS | chr1 | 15398763 | XP_022315236.1 | dual 3'%2C5'-cyclic-AMP and -GMP phosphodiesterase 11-like isoform X2 | 3 x 10^-99^ | GO:0003674;GO:0007165;GO:0034641;GO:0044281 | EC:3.1.4.17 | 3',5'-cyclic-nucleotide phosphodiesterase |
| MS | chr1 | 44427940 | XP_022345317.1 | uncharacterized protein LOC111137902 | 3 x 10^-99^ |  |  |  |
| MS | chr1 | 44427945 | XP_022345317.1 | uncharacterized protein LOC111137902 | 3 x 10^-99^ |  |  |  |
| MS | chr1 | 44427962 | XP_022345317.1 | uncharacterized protein LOC111137902 | 3 x 10^-99^ |  |  |  |
| MS | chr1 | 54789017 | XP_022291696.1 | rac guanine nucleotide exchange factor B-like isoform X3 | 3 x 10^-61^ | GO:0003674 |  |  |
| MS | chr2 | 34639155 | XP_022306860.1 | farnesyl pyrophosphate synthase-like | 7 x 10^-13^ | GO:0006629;GO:0009058;GO:0016765 | EC:2.5.1.10;EC:2.5.1.1 | (2E,6E)-farnesyl diphosphate synthase;Dimethylallyltranstransferase |
| MS | chr3 | 31340804 | XP_022322746.1 | uncharacterized protein LOC111124176 | 3 x 10^-99^ |  |  |  |
| MS | chr3 | 54992845 | XP_022298078.1 | heat shock 70 kDa protein 12A-like | 9 x 10^-18^ |  |  |  |
| MS | chr4 | 9362131 | XP_022331168.1 | forkhead box protein N3-like | 3 x 10^-99^ | GO:0003677;GO:0003700;GO:0005622;GO:0009058;GO:0032991;GO:0034641 |  |  |
| MS | chr4 | 14405539 | XP_022332702.1 | coiled-coil domain-containing protein 171-like isoform X4 | 1 x 10^-22^ |  |  |  |
| MS | chr4 | 14405640 | XP_022332702.1 | coiled-coil domain-containing protein 171-like isoform X4 | 1 x 10^-22^ |  |  |  |
| MS | chr4 | 14408152 | XP_022304022.1 | uncharacterized protein LOC111111367 isoform X3 | 0.001 |  |  |  |
| MS | chr4 | 18073877 | XP_022334824.1 | probable G-protein coupled receptor CG31760 | 7 x 10^-83^ | GO:0003674;GO:0005575;GO:0007165 |  |  |
| MS | chr4 | 43336624 | XP_022286782.1 | uncharacterized protein LOC111099683 | 9 x 10^-18^ |  |  |  |
| MS | chr4 | 56226525 | XP_022339207.1 | uncharacterized protein LOC111134466 | 2 x 10^-12^ | GO:0003674 |  |  |
| MS | chr5 | 17555674 | XP_022338407.1 | LOW QUALITY PROTEIN: cysteine-rich venom protein 1-like | 4 x 10^-10^ | GO:0005576 |  |  |
| MS | chr5 | 17555750 | XP_022338407.1 | LOW QUALITY PROTEIN: cysteine-rich venom protein 1-like | 1 x 10^-10^ | GO:0005576 |  |  |
| MS | chr5 | 48915994 | XP_02289483.1 | E3 ubiquitin-protein ligase TRIM71-like | 6 x 10^-14^ | GO:0043167 |  |  |
| MS | chr5 | 48916022 | XP_022334799.1 | proton-coupled folate transporter-like | 2 x 10^-12^ | GO:0003674;GO:0005575;GO:0055085 |  |  |
| MS | chr5 | 61694641 | XP_022336786.1 | eukaryotic translation initiation factor 2A-like | 4 x 10^-47^ | GO:0005840;GO:0008135 |  |  |
| MS | chr5 | 61694680 | XP_022336786.1 | eukaryotic translation initiation factor 2A-like | 4 x 10^-47^ |  |  |  |
| MS | chr5 | 61694719 | XP_022336786.1 | eukaryotic translation initiation factor 2A-like | 4 x 10^-47^ |  |  |  |
| MS | chr5 | 61804560 | XP_022346086.1 | uncharacterized protein LOC111138422 | 3 x 10^-99^ |  |  |  |
| MS | chr5 | 61804618 | XP_022346086.1 | uncharacterized protein LOC111138422 | 3 x 10^-99^ |  |  |  |
| MS | chr5 | 62335542 | XP_022340859.1 | Na(+)/H(+) exchanger beta-like | 3 x 10^-99^ | GO:0005575;GO:0022857;GO:0042592 |  |  |
| MS | chr5 | 62781915 | XP_022335244.1 | ras-related protein Rab-10-like | 1 x 10^-42^ | GO:0003924;GO:0043167 | EC:3.6.1.15 | Nucleoside-triphosphate phosphatase |
| MS | chr5 | 65487728 | XP_022308685.1 | sialin-like | 1 x 10^-4^ | GO:0005575;GO:0055085 |  |  |
| MS | chr5 | 65988573 | XP_022335403.1 | uncharacterized protein LOC111132056 isoform X2 | 3 x 10^-99^ |  |  |  |
| MS | chr5 | 66359928 | XP_022342097.1 | myoferlin-like isoform X19 | 1 x 10^-41^ | GO:0005575 |  |  |
| MS | chr5 | 68753201 | XP_022338241.1 | centrosomal protein of 19 kDa-like | 2 x 10^-71^ |  |  |  |
| MS | chr5 | 68843212 | XP_022341851.1 | ecto-NOX disulfide-thiol exchanger 2-like | 3 x 10^-4^ | GO:0005886;GO:0016491 |  |  |
| MS | chr5 | 68843434 | XP_022341851.1 | ecto-NOX disulfide-thiol exchanger 2-like | 5 x 10^-78^ | GO:0005886;GO:0016491 |  |  |
| MS | chr5 | 68843481 | XP_022341851.1 | ecto-NOX disulfide-thiol exchanger 2-like | 2 x 10^-69^ | GO:0005886;GO:0016491 |  |  |
| MS | chr5 | 71392872 | XP_022343889.1 | uncharacterized protein LOC111136978 | 3 x 10^-99^ |  |  |  |
| MS | chr5 | 71392909 | XP_022343889.1 | uncharacterized protein LOC111136978 | 3 x 10^-99^ |  |  |  |
| MS | chr5 | 72787167 | XP_022338241.1 | centrosomal protein of 19 kDa-like | 3 x 10^-99^ |  |  |  |
| MS | chr5 | 73089667 | XP_022339622.1 | clathrin heavy chain 2-like | 3 x 10^-11^ | GO:0005198;GO:0005794;GO:0005886;GO:0016192;GO:0031410;GO:0032991 |  |  |
| MS | chr5 | 73332073 | XP_022338483.1 | probable G-protein coupled receptor 139 | 3 x 10^-99^ | GO:0003674;GO:0005575;GO:0007165 |  |  |
| MS | chr5 | 75161604 | XP_022338224.1 | ATP-dependent DNA helicase DDX11-like | 9 x 10^-37^ | GO:0003677;GO:0004386;GO:0005694;GO:0016887;GO:0034641;GO:0043167 | EC:3.6.1.3;EC:3.6.1.15 | Adenosinetriphosphatase;Nucleoside-triphosphate phosphatase |
| MS | chr5 | 76666894 | XP_022336144.1 | uncharacterized protein LOC111132609 | 3 x 10^-99^ | GO:0007165;GO:0019899 |  |  |
| MS | chr5 | 77131180 | XP_022345121.1 | RING finger protein 145-like | 1 x 10^-29^ | GO:0043167 |  |  |
| MS | chr6 | 31381216 | XP_022288369.1 | putative transferase CAF17 homolog%2C mitochondrial | 3 x 10^-5^ | GO:0003674 |  |  |
| MS | chr6 | 31831633 | XP_022339677.1 | LOW QUALITY PROTEIN: sterile alpha and TIR motif-containing protein 1-like | 5 x 10^-46^ | GO:0002376;GO:0003674;GO:0006950;GO:0007165 |  |  |
| MS | chr6 | 32082204 | XP_022288785.1 | probable ATP-dependent RNA helicase DDX58 | 3 x 10^-99^ | GO:0043167 |  |  |
| MS | chr6 | 32397315 | XP_022286836.1 | sucrase-isomaltase%2C intestinal-like | 3 x 10^-11^ | GO:0005975;GO:0016798 |  |  |
| MS | chr6 | 32397318 | XP_022286836.1 | sucrase-isomaltase%2C intestinal-like | 7 x 10^-13^ | GO:0005975;GO:0016798 |  |  |
| MS | chr6 | 32397624 | XP_022286836.1 | sucrase-isomaltase%2C intestinal-like | 4 x 10^-10^ | GO:0005975;GO:0016798 |  |  |
| MS | chr6 | 32397627 | XP_022286836.1 | sucrase-isomaltase%2C intestinal-like | 4 x 10^-10^ | GO:0005975;GO:0016798 |  |  |
| MS | chr6 | 37347819 | XP_022286861.1 | cell division cycle protein 20 homolog | 3 x 10^-49^ | GO:0006464;GO:0009056;GO:0030234 |  |  |
| MS | chr6 | 37347945 | XP_022286861.1 | cell division cycle protein 20 homolog | 4 x 10^-79^ | GO:0006464;GO:0009056;GO:0030234 |  |  |
| MS | chr6 | 42918409 | XP_022287884.1 | von Willebrand factor D and EGF domain-containing protein-like | 2 x 10^-57^ |  |  |  |
| MS | chr8 | 37850455 | XP_022292924.1 | uncharacterized protein LOC111103756 isoform X3 | 3 x 10^-4^ |  |  |  |
| MS | chr8 | 73448225 | XP_022300205.1 | uncharacterized protein LOC111108532 | 7 x 10^-45^ | GO:0003674;GO:0005576;GO:0008150 |  |  |
| MS | chr9 | 80959479 | XP_022310252.1 | uncharacterized protein LOC111115712 | 3 x 10^-99^ |  |  |  |
| MS | chr9 | 97420374 | XP_022326476.1 | sodium- and chloride-dependent glycine transporter 2-like | 5 x 10^-8^ | GO:0005575;GO:0022857 |  |  |
| MinWT | chr1 | 3055672 | XP_022332006.1 | exportin-1-like | 4 x 10^-28^ | GO:0006810;GO:0007165;GO:0019899 |  |  |
| MinWT | chr1 | 40326822 | XP_022328043.1 | ufm1-specific protease 2-like | 7 x 10^-13^ |  |  |  |
| MinWT | chr2 | 13041257 | XP_022320827.1 | polygalacturonase 1 beta-like protein 1 | 4 x 10^-54^ |  |  |  |
| MinWT | chr2 | 13041441 | XP_022320827.1 | polygalacturonase 1 beta-like protein 1 | 4 x 10^-10^ |  |  |  |
| MinWT | chr2 | 22206407 | XP_022335562.1 | inosine-uridine preferring nucleoside hydrolase-like isoform X3 | 8 x 10^-19^ |  |  |  |
| MinWT | chr2 | 23025759 | XP_022319288.1 | ABC transporter G family member 14-like | 7 x 10^-13^ | GO:0005575;GO:0016887;GO:0043167 | EC:3.6.1.3;EC:3.6.1.15 | Adenosinetriphosphatase;Nucleoside-triphosphate phosphatase |
| MinWT | chr2 | 43516534 | XP_022317650.1 | myosin heavy chain%2C striated muscle-like isoform X7 | 6 x 10^-14^ | GO:0005856;GO:0008092;GO:0032991;GO:0043167 | EC:3.6.1.15 | Nucleoside-triphosphate phosphatase |
| MinWT | chr2 | 43516558 | XP_022317650.1 |  | 5 x 10^-27^ |  |  |  |
| MinWT | chr2 | 43516656 | XP_022317650.1 |  | 7 x 10^-26^ |  |  |  |
| MinWT | chr2 | 43516819 | XP_022317650.1 |  | 4 x 10^-35^ |  |  |  |
| MinWT | chr2 | 43516841 | XP_022317650.1 |  | 5 x 10^-15^ |  |  |  |
| MinWT | chr3 | 20195903 | XP_022327522.1 | acetylcholine receptor subunit gamma-like isoform X2 | 2 x 10^-31^ | GO:0005575;GO:0007165;GO:0022857 |  |  |
| MinWT | chr3 | 34216538 | XP_022335267.1 | type I iodothyronine deiodinase-like | 3 x 10^-5^ | GO:0016491 | EC:1.97.1.10 | Other oxidoreductases |
| MinWT | chr3 | 58782357 | XP_022327688.1 | coiled-coil domain-containing protein 22 homolog | 8 x 10^-44^ |  |  |  |
| MinWT | chr4 | 53244996 | XP_022301770.1 | globin-like | 6 x 10^-33^ | GO:0003674 |  |  |
| MinWT | chr5 | 62781909 | XP_022305585.1 | extracellular tyrosine-protein kinase PKDCC-like | 1 x 10^-53^ | GO:0006464;GO:0016301;GO:0043167 |  |  |
| MinWT | chr5 | 67495190 | XP_022342514.1 | uncharacterized protein LOC111136157 | 3 x 10^-4^ | GO:0003674 |  |  |
| MinWT | chr5 | 69770327 | XP_022343647.1 | acid sphingomyelinase-like phosphodiesterase 3b | 1 x 10^-61^ | GO:0003674 |  |  |
| MinWT | chr5 | 69858610 | XP_022344414.1 | ankyrin repeat domain-containing protein 34B-like isoform X2 | 5 x 10^-46^ | GO:0003674 |  |  |
| MinWT | chr5 | 73057556 | XP_022341265.1 | protein FAM124A-like isoform X2 | 1 x 10^-10^ |  |  |  |
| MinWT | chr6 | 20902475 | XP_022286824.1 | uncharacterized protein LOC111099704 | 1 x 10^-21^ |  |  |  |
| MinWT | chr6 | 32458744 | XP_022323128.1 | protein wech-like | 1 x 10^-23^ | GO:0043167 |  |  |
| MinWT | chr6 | 37113676 | XP_022288270.1 | propionyl-CoA carboxylase alpha chain%2C mitochondrial-like isoform X1 | 1 x 10^-28^ | GO:0043167 |  |  |
| MinWT | chr6 | 37113801 | XP_022288270.1 | propionyl-CoA carboxylase alpha chain%2C mitochondrial-like isoform X1 | 3 x 10^-55^ | GO:0043167 |  |  |
| MinWT | chr6 | 37113888 | XP_022288270.1 | propionyl-CoA carboxylase alpha chain%2C mitochondrial-like isoform X1 | 1 x 10^-10^ | GO:0043167 |  |  |
| MinWT | chr6 | 31132382 | XP_022300026.1 | receptor-interacting serine/threonine-protein kinase 4-like isoform X3 | 5 x 10^-8^ | GO:0003674 |  |  |
| MpH | chr1 | 7959255 | XP_022306356.1 | uncharacterized protein LOC111112818 | 4 x 10^-13^ | GO:0003674 |  |  |
| MpH | chr1 | 13636056 | XP_022287164.1 | uncharacterized protein LOC111099929 isoform X8 | 6 x 10^-13^ |  |  |  |
| MpH | chr1 | 15360460 | XP_022286854.1 | MAM and LDL-receptor class A domain-containing protein 1-like isoform X2 | 4 x 10^-16^ | GO:0003674;GO:0005575 |  |  |
| MpH | chr1 | 40736489 | XP_022328667.1 | patatin-like phospholipase domain-containing protein 2 | 3 x 10^-99^ | GO:0003674;GO:0006629;GO:0009056 |  |  |
| MpH | chr1 | 42499514 | XP_022318158.1 | uncharacterized protein LOC111121258 isoform X2 | 2 x 10^-38^ | GO:0006464;GO:0016301;GO:0043167 | EC:2.7.10 | Transferring phosphorus-containing groups |
| MpH | chr1 | 42499517 | XP_022318158.1 | uncharacterized protein LOC111121258 isoform X2 | 2 x 10^-38^ | GO:0006464;GO:0016301;GO:0043167 | EC:2.7.10 | Transferring phosphorus-containing groups |
| MpH | chr3 | 18198085 | XP_022298976.1 | serine/threonine-protein phosphatase 6 regulatory ankyrin repeat subunit C-like | 1 x 10^-16^ | GO:0003674 |  |  |
| MpH | chr4 | 14204746 | XP_022337733.1 | laccase-4-like | 8 x 10^-19^ | GO:0016491;GO:0043167 |  |  |
| MpH | chr4 | 38954995 | XP_022330772.1 | neuropeptide Y receptor type 6-like | 2 x 10^-19^ | GO:0003674;GO:0005575;GO:0007165 |  |  |
| MpH | chr5 | 36513467 | XP_022298711.1 | spermatogenesis-associated protein 20-like isoform X3 | 2 x 10^-57^ | GO:0003674 |  |  |
| MpH | chr5 | 69642286 | XP_022327818.1 | N-acetylneuraminate 9-O-acetyltransferase-like isoform X3 | 9 x 10^-12^ |  |  |  |
| MpH | chr5 | 73295567 | XP_022345819.1 | protein draper-like | 3 x 10^-4^ |  |  |  |
| MpH | chr6 | 43149879 | XP_022289320.1 | Kv channel-interacting protein 4-like isoform X16 | 1 x 10^-60^ | GO:0043167 |  |  |
| MpH | chr6 | 50486734 | XP_022303254.1 | uncharacterized protein LOC111110887 | 2 x 10^-6^ | GO:0043167 |  |  |
| MpH | chr7 | 13535195 | XP_022294557.1 | uncharacterized protein LOC111104745 | 2 x 10^-51^ |  |  |  |
| MpH | chr7 | 26351220 | XP_022288690.1 | E3 ubiquitin-protein ligase TRIM71-like | 7 x 10^-74^ | GO:0043167 |  |  |
| MpH | chr7 | 46041405 | XP_022291790.1 | uncharacterized protein LOC111103076 | 1 x 10^-9^ |  |  |  |
| MinpH | chr1 | 19401881 | XP_022292455.1 | angiopoietin-related protein 7-like | 3 x 10^-99^ |  |  |  |
| MinpH | chr2 | 22108745 | XP_022306911.1 | receptor-type tyrosine-protein phosphatase epsilon-like isoform X3 | 2 x 10^-7^ | GO:0006464;GO:0006520;GO:0016791 | EC:3.1.3.16;EC:3.1.3.48 | Protein-serine/threonine phosphatase;Protein-tyrosine-phosphatase |
| MinpH | chr3 | 61872611 | XP_022320666.1 | LOW QUALITY PROTEIN: low-density lipoprotein receptor-related protein 2-like | 5 x 10^-65^ | GO:0043167 |  |  |
| MinpH | chr3 | 67869114 | XP_022295570.1 | E3 ubiquitin-protein ligase arih1-like | 3 x 10^-30^ | GO:0003674;GO:0006464 |  |  |
| MinpH | chr5 | 66381543 | XP_022343779.1 | uncharacterized protein LOC111136903 isoform X4 | 3 x 10^-61^ |  |  |  |
| MinpH | chr5 | 67489624 | XP_022339347.1 | lysosome-associated membrane glycoprotein 1-like | 3 x 10^-99^ | GO:0005575 |  |  |
| MinpH | chr5 | 67489647 | XP_022339347.1 | lysosome-associated membrane glycoprotein 1-like | 3 x 10^-99^ | GO:0005575 |  |  |
| MinpH | chr5 | 69666077 | XP_022343230.1 | transcription factor Sox-2-like | 1 x 10^-4^ | GO:0003677;GO:0005634;GO:0009058;GO:0034641 |  |  |
| MinpH | chr5 | 69666199 | XP_022343230.1 | transcription factor Sox-2-like | 1 x 10^-4^ | GO:0003677;GO:0005634;GO:0009058;GO:0034641 |  |  |
| MinpH | chr5 | 69666200 | XP_022343230.1 | transcription factor Sox-2-like | 1 x 10^-4^ | GO:0003677;GO:0005634;GO:0009058;GO:0034641 |  |  |
| MinpH | chr5 | 71889468 | XP_022336578.1 | zinc finger protein ZIC 4-like | 3 x 10^-99^ | GO:0003674 |  |  |
| MinpH | chr5 | 77093544 | XP_022332716.1 | carcinoembryonic antigen-related cell adhesion molecule 5-like | 5 x 10^-8^ | GO:0005575;GO:0007155 |  |  |
| MinpH | chr6 | 30876102 | XP_022335446.1 | uncharacterized protein LOC111132081 | 4 x 10^-9^ | GO:0005575 |  |  |
| MinpH | chr6 | 33055474 | XP_022286676.1 | uncharacterized protein LOC111099612 isoform X2 | 6 x 10^-20^ | GO:0003674;GO:0007165 |  |  |
| MinpH | chr6 | 33055488 | XP_022286676.1 | uncharacterized protein LOC111099612 isoform X2 | 2 x 10^-27^ | GO:0003674;GO:0007165 |  |  |
| MinpH | chr6 | 33055715 | XP_022286676.1 | uncharacterized protein LOC111099612 isoform X2 | 5 x 10^-59^ | GO:0003674;GO:0007165 |  |  |
| MinpH | chr8 | 46972315 | XP_022298284.1 | xanthine dehydrogenase/oxidase-like isoform X2 | 3 x 10^-43^ | GO:0016491;GO:0043167 |  |  |
| MinpH | chr8 | 48715222 | XP_022298276.1 | serine/threonine-protein phosphatase 6 regulatory ankyrin repeat subunit A-like | 2 x 10^-27^ | GO:0003674 |  |  |
| MinpH | chr8 | 60614519 | XP_022321462.1 | uncharacterized protein LOC111123437 | 2 x 10^-25^ | GO:0003677;GO:0006259 |  |  |
| MinpH | chr9 | 88584638 | XP_022339700.1 | carbonic anhydrase 2-like isoform X2 | 4 x 10^-9^ | GO:0016829;GO:0043167;GO:0044281 | EC:4.2.1.1 | Carbonic anhydrase |

**Table S5.** Effective population size (N_e_) for *C. virginica* sampling site from this study (Chesapeake Bay) and from Bernatchez et al., (2019) (Canada)

| **Sites (Canada)** | **N** | **N_e_ (CI) 0.20** | **Sites (Chesapeake Bay)** | **N_e_ (CI) 0.20** |
| --- | --- | --- | --- | --- |
| BOU | 40 | 3983.6 (2541.7, 9167.7) | HCR1 | 71.1 (38.5, 244.2) |
| COC | 38 | 236.8 (69.1, ∞) | HCR2 | 155.5 (115.5, 232.9) |
| CRB | 33 | 2240.7 (1772.3, 3043.2) | HCR4 | 325.8 (225.3, 574.4) |
| CRQ | 40 | 5574.3 (3745.6, 10872.1) | HCS | 67.3 (32.9, 310.8) |
| INK | 38 | 7071.7 (4321.2, 19386.6) | HCW | 75.2 (43.3, 188.7) |
| MAL | 35 | 3017.8 (2249.9, 4575.8) | LC | 501.4 (362.3, 804.5) |
| MIR | 37 | 6297.4 (4078.9, 13777.6) | TB | 141.4 (92.1, 278.0) |
| MIS | 39 | 6254.2 (3959.3, 14834.4) | BR | 382.8 (188.2, 12461.9) |
| RIC | 39 | 1180.5 (832.4, 2017.6) | TS | 123.6 (44.5, ∞) |
| SHD | 39 | 2952.9 (2196.1, 4499.7) | JR | 346.3 (216.2, 819.4) |
| SHM | 38 | 2917.6 (1743, 8855.4) | W | 193.3 (118.3, 480.3) |
| SSI | 30 | 1917.6 (1503.5, 2644) |  |  |
| TAB | 40 | 5966.3 (4119.9, 10796) |  |  |

Abbreviations of Chesapeake Bay sampling sites are presented in Table 1.

Abbreviations of Canada sampling sites are presented in Bernatchez et al. (2018)

**Table S6.** Mean, minimum, and maximum annual values for environmental variables used in redundancy analysis (RDA) for salinity (ppt), DO (mg/L), temperature (°C) for each of the Chesapeake Bay sampling sites. SD represents the standard deviation for each environmental variable across the sampling sites.

|  | Mean Salinity (ppt) | Minimum Salinity (ppt) | Mean DO (mg/L) | Minimum DO (mg/L) | Minimum Temperature (°C) | Maximum Temperature (°C) | Mean pH | Minimum pH |
| --- | --- | --- | --- | --- | --- | --- | --- | --- |
| HCR1 | 12.3 | 6.7 | 8.79 | 3.93 | 1.03 | 33.57 | 7.99 | 7.14 |
| HCR2 | 12.52 | 6.64 | 8.59 | 2.91 | 2.52 | 29.92 | 7.92 | 7.26 |
| HCR4 | 12.3 | 6.7 | 8.79 | 3.93 | 1.03 | 33.57 | 7.99 | 7.14 |
| HCS | 12.52 | 6.64 | 8.59 | 2.91 | 2.52 | 29.92 | 7.92 | 7.26 |
| HCW | 12.52 | 6.64 | 8.59 | 2.91 | 2.52 | 29.92 | 7.92 | 7.26 |
| LC | 13.27 | 13.27 | 8.37 | 6.6 | 3 | 28.2 | 7.99 | 7.3 |
| TB | 10.55 | 5.45 | 8.72 | 5.3 | 3.6 | 28.2 | 7.79 | 7.3 |
| BR | 16.38 | 12 | 7.5 | 2.65 | 2.9 | 27.1 | 7.97 | 7.3 |
| JR | 17.35 | 13.27 | 8.03 | 6.01 | 2.6 | 28.65 | 7.86 | 7.74 |
| TS | 17.39 | 9.53 | 9.33 | 3.9 | 4.2 | 27 | 7.99 | 7.7 |
| W | 28.57 | 20.48 | 6.9 | 3.67 | 7.86 | 29.91 | 7.89 | 7.49 |
| SD | 4.80 | 4.38 | 0.64 | 1.28 | 1.68 | 2.13 | 0.06 | 0.19 |

Abbreviations of Chesapeake Bay sampling sites are presented in Table 1.
