# Supplementary material for: Genome‐wide analysis of natural and restored eastern oyster populations reveals local adaptation and positive impacts of planting frequency and broodstock number
Source: Evol Appl. 2021 Dec 7;15(1):40–59. doi: 10.1111/eva.13322 (PMC8792482; doi:10.1111/eva.13322)
Supplement: Supplementary file 2 — Supplementary Material [file EVA-15-40-s001.docx]

**Supporting Information**

Includes Supplementary Methods S1-S5

**Supplementary Methods**

**S1. Restriction enzyme digestion**

Genomic DNA was extracted using the E.Z.N.A.® Tissue DNA Kit (Omega-Biotek, Norcross, GA), following the protocol for animal tissues with RNAse-A treatment following manufacturer instructions. DNA samples were normalized to a concentration of 50 ng/µL and transferred to 96-well plates. Enzymatic digestion was performed in 50 µl volumes by digesting ~500 ng of normalized genomic DNA from each sample in 1X cutsmart® buffer with 20 units of EcoRI-HF® (New England Biolabs, R3101S) and 20 units of SphI-HF® (New England Biolabs, R3182S) restriction enzymes. Incubation was carried out at 37^o^C for 3 hours. Digests were quantified using the Qubit dsDNA BR Assay Kit (Life Technologies) on a Qubit v2.0 (Life Technologies) to standardize DNA to 250 ng for P1 and P2 FLEX adaptor ligation (New England Biolabs). Samples were then pooled into ‘index’ libraries consisting of 48 individuals each and size selected which provides control of the number of homologous fragments returned from digestion. Fragments were selected in the range of 250-800 bp in length using a double-size selection with Ampure XP beads and 20% PEG. Index-specific identifiers were added to each library index (pool of 48 samples) using 12 PCR cycles.

Paired-end 250-bp sequencing was conducted on the Illumina HiSeq 2500 platform at Genewiz, Inc. (South Plainfield, NJ) spread across two and a half lanes.

**S2. SNP discovery and filtering**

Read quality was evaluated using FastQC v.0.11.5 (Andrews, 2010). Raw sequences were demultiplexed using the *process_radtags* component of Stacks v.2.0 (Catchen et al. 2013). Samples underwent simultaneous mapping, SNP discovery, and genotyping using the *dDocent* pipeline v. 2.7.7 (Puritz et al. 2014) with default settings unless otherwise noted. Trimmed reads were directly mapped to the *C. virginica* genome (NCBI Bioprojects: PRJNA379157 and PRJNA376014, accession numbers: NC_007175.2 and NC_035780.1 – NC_035789.1) using the MEM algorithm of Burrows-Wheeler Aligner (BWA; Li and Durbin 2009) with parameters A (match score), B (mismatch score), and O (gap penalty) set to 1,2, and 5, respectively which are appropriate for genomic data of marine species (Puritz, unpublished data). Freebayes v1.2.0-dirty (Garrison and Marth 2012) was used to obtain raw variant calls and SNP genotypes.

SNP loci were filtered using VCFtools (Danecek et al. 2011). Loci were removed that had a minor allele count of less than 3, a PHED quality score of less than 20, and a call rate of less than 50%. To remove individuals that did not sequence well, genotypes with less than 5 reads were recorded as missing. Seventy-nine individuals (out of 570) were removed for having more than 50% missing data using the script filter_missing_ind.sh (https://github.com/jpuritz/dDocent/blob/master/scripts/filter_missing_ind.sh). Loci called in less than 90% of individuals overall with a minor allele frequency of less than 5% were removed. Loci were then removed that were not called in 75% of individuals in any one population using the script pop_missing_filter.sh (https://github.com/jpuritz/dDocent/blob/master/scripts/pop_missing_filter.sh). Variant calls were filtered using the dDocent_filters script (<https://github.com/jpuritz/dDocent/blob/master/scripts/dDocent_filters>); loci were removed if average allele balance at heterozygous genotypes was less than 25%, if quality sum of reference or alternative alleles was 0, if quality score was less than half of the total depth, if the ratio between mean mapping quality of the alternate and reference alleles was less than 0.9 or more than 1.05, if the majority of reads did not come from only one read orientation, based on the status of properly paired reads and if the quality score is less than two times the depth. Variant calls were then decomposed into SNP and INDEL calls using vcflib. Using the script filter_hwe_by_pop.pl, SNPs were removed that had a P value of less than 0.001 in at least 50% of the populations (<https://github.com/jpuritz/dDocent/blob/master/scripts/filter_hwe_by_pop.pl)>. Using VCFtools, SNPs were filtered to only include loci with 2 alleles. Next, genotypes of five individuals, intentionally duplicated in different RAD libraries, were compared to look for genotype discordance consistent with systematic genotyping error. Loci which exhibited genotype differences across more than one set of replicates were discarded, and the individual from each set of duplicates that contained the most missing data across loci was removed from the data set.

Finally, SNPs were pruned in PLINK v1.90b6.18 (Chang et al. 2015) to remove those exhibiting high linkage disequilibrium defined by using a 5kb window, sliding 5 SNPs, and remove a SNP from all pairs with r^2^>0.5.

### S3. Outlier detection and defining datasets

Three outlier detection methods with different underlying models were used to partition SNPs into groups of putatively neutral versus SNPs putatively under directional selection: Bayescan (v.2.1) (Foll and Gaggiotti 2008) with prior model of 10,000 following recommendations of Lotterhos and Whitlock (2015), 10,000 iterations, a burn-in of 200,000 steps and a false discovery rate (FDR) of 0.05; OutFLANK (v.0.2) (Whitlock and Lotterhos 2015) with default options (LeftTrimFraction=0.05, RightTrimFraction=0.05, Hmin=0.1, 11) and a q-threshold of 0.05, and pcadapt (v.4.1.0) with a q-value threshold of 0.05 (Luu et al. 2017). For Bayescan and OutFLANK analyses, individuals were grouped into populations by sampling site. For pcadapt, multiple values of K (principal components) were tested, and the final number of PC axes retained was determined by visual inspection of the scree plot. Multiple methods were utilized to minimize the occurrence of false positives as recommended by Hoban et al. (2016).

### S4. Genotype-environment associations

When compared to differentiation-based outlier methods, RDA can detect even weak multi-locus signatures of selection for multiple environmental variables (Rellstab et al. 2015, Forester et al. 2018). Environmental variables considered to be important for oysters were downloaded (three related to salinity and temperature, and two related to dissolved oxygen (DO) and pH from 2014-2018). The environmental parameters used were annual minimum, mean, and maximum salinity and water temperature, and annual mean and minimum DO and pH, at a given site. Some buoys had continuous monitoring (data every 15 minutes; Table 1) while others sampled only two or four times per month, so the minimum resolution available for all sites, sampling twice per month over four years, was used (N=96 for each variable). Missing values for environmental data were replaced with the median using the R package RANDOMFOREST v.4.6-14 (Liaw and Wiener 2001) (96 data points for each environmental parameter).

For pre-analysis data filtering of the full dataset, correlations between environmental variables were evaluated using the Pearson correlation coefficient and when two variables were highly correlated (|r|$\geq$0.7), only one variable was retained. The resulting set of variables included one variable related to temperature (annual minimum water temperature), one related to salinity (annual mean salinity), two related to pH (annual mean and minimum pH), and one related to DO (annual minimum DO). For the inner Bay dataset, the resulting set of variables included one variable related to temperature (annual maximum water temperature), one related to salinity (annual minimum salinity), one related to pH (annual minimum pH), and two related to DO (annual mean and minimum DO). RDA requires complete data frames, so missing genotype data was imputed by using the most common genotype across individuals (Forester et al. 2018).

### S5. Effect of environmental variables and geography on genetic variation

RDA is a useful multivariate regression technique when running regression analyses with multivariate predictors (space and environment) and multivariate responses (here, allele frequencies of SNPs). For this analysis, the thinned neutral dataset (4,641 SNPs) and the putatively adaptive dataset (see Results) was used with environmental variables and spatial variables (X and Y coordinates). Spatial variables based on the x-y coordinates were defined using the principal coordinates of neighborhood matrices (PCNMs), also known as Moran’s eigenvector maps (MEM) using the *pcnm* function in vegan. Half of the PCNM variables with positive eigenvalues were retained, which has been suggested in similar contexts (Manel et al. 2012, Fitzpatrick and Keller 2015).

RDA was conducted on the neutral and putative outlier datasets separately to assess the influence of environmental variables and geographic distance on observed patterns of genetic variation (Borcard et al. 1992, Liu 1997, Legendre and Fortin 2010, Bie et al. 2012). Genetic data and environmental variables were compared in a partial RDA specifying geography (PCNMs) as a third conditioned matrix so that the analysis conditions on geographic location. To assess the correlation between genotype and each spatial/environmental variable, an analysis of variance (ANOVA) was performed with 1,000 permutations one variable at a time and variables with p $\leq$0.1 were retained. Then, the variance inflation factor (VIF; vif.cca function implemented in vegan) was calculated to evaluate multicollinearity of all retained variables (Hair et al*.* 1995; Zuur et al. 2010; James et al. 2013) variables with VIF>=10 were excluded (Hair et al. 1995). The ordistep function from the R package vegan was used to select the most important explanatory variables among those retained. The final RDA was assessed using an ANOVA and marginal ANOVAs (1,000 permutations) to assess the contribution of each environmental variable. Next, to explain how much of the genetic variation in *C. virginica* is uniquely explained by environmental variables, how much is uniquely explained by geography, and how much is due to the combined effect of the two, variance components of the RDA were partitioned by running 3 models: a full model with environmental and geographic variables; a partial model in which geography explains genetic data conditioned on important environmental variables; a partial model in which important environmental variables explain genetic data conditioned on geography. This analysis allowed for distinguishing between how much of the total explainable neutral and adaptive variance was due to the environment (after removing geographical effects), how much was due to geography (after removing environmental effects), and how much was due to the joint effect of both factors.

**References**

Andrews S. 2010. FastQC: a quality control tool for high throughput sequence data. Available online at: <http://www.bioinformatics.babraham.ac.uk/projects/fastqc>

Bie, T., L. Meester, L. Brendonck, K. Martens, B. Goddeeris, D. Ercken, H. Hampel, L. Denys, L. Vanhecke, K. Gucht, J. Wichelen, W. Vyverman, and S. A. J. Declerck. 2012. Body size and dispersal mode as key traits determining metacommunity structure of aquatic organisms. Ecology Letters 15:740–747.

Borcard, D., P. Legendre, and P. Drapeau. 1992. Partialling out the spatial component of ecological variation. Ecology 73:1045–1055.

Catchen, J., P. A. Hohenlohe, S. Bassham, A. Amores, and W. A. Cresko. 2013. Stacks: an analysis tool set for population genomics. Molecular Ecology 22:3124–3140.

Chang, C. C., C. C. Chow, L. C. Tellier, S. Vattikuti, S. M. Purcell, and J. J. Lee. 2015. Second-generation PLINK: rising to the challenge of larger and richer datasets. GigaScience 4:7.

Danecek, P., A. Auton, G. Abecasis, C. A. Albers, E. Banks, M. A. DePristo, R. E. Handsaker, G. Lunter, G. T. Marth, S. T. Sherry, G. McVean, and R. Durbin. 2011. The variant call format and VCFtools. Bioinformatics 27:2156–2158.

Fitzpatrick, M. C., and S. R. Keller. 2015. Ecological genomics meets community-level modelling of biodiversity: mapping the genomic landscape of current and future environmental adaptation. Ecology Letters 18:1–16.

Foll, M., and O. Gaggiotti. 2008. A Genome-Scan Method to Identify Selected Loci Appropriate for Both Dominant and Codominant Markers: A Bayesian Perspective. Genetics 180:977–993.

Forester, B. R., J. R. Lasky, H. H. Wagner, and D. L. Urban. 2018. Comparing methods for detecting multilocus adaptation with multivariate genotype–environment associations. Molecular Ecology 27:2215–2233.

Garrison, E., and G. Marth. 2012. Haplotype-based variant detection from short-read sequencing. arXiv:1207.3907 [q-bio].

Hair, J. F., Jr., R.E. Anderson, R.L. Tatham, and W.C. Black. 1995. Multivariate Data Analysis, 3rd ed, Macmillan Publishing Company, New York.

Hoban, S., J. L. Kelley, K. E. Lotterhos, M. F. Antolin, G. Bradburd, D. B. Lowry, M. L. Poss, L. K. Reed, A. Storfer, and M. C. Whitlock. 2016. Finding the Genomic Basis of Local Adaptation: Pitfalls, Practical Solutions, and Future Directions. The American Naturalist 188:379–397.

James, G., D. Witten, T. Hastie, and R. Tibshirani, editors. 2013. An introduction to statistical learning: with applications in R. Springer, New York.

Legendre, P., and M.-J. Fortin. 2010. Comparison of the Mantel test and alternative approaches for detecting complex multivariate relationships in the spatial analysis of genetic data. Molecular Ecology Resources 10:831–844.

Li, H., and R. Durbin. 2009. Fast and accurate short read alignment with Burrows–Wheeler transform. Bioinformatics 25:1754–1760.

Liaw, A., and M. Wiener. 2001. Classification and Regression by RandomForest. Forest 23.

Liu, Q. 1997. Variation partitioning by partial redundancy analysis (RDA). Environmetrics 8:75–85.

Lotterhos, K. E., and M. C. Whitlock. 2015. The relative power of genome scans to detect local adaptation depends on sampling design and statistical method. Molecular Ecology 24:1031–1046.

Manel, S., F. Gugerli, W. Thuiller, N. Alvarez, P. Legendre, R. Holderegger, L. Gielly, P. Taberlet, and I. Consortium. 2012. Broad-scale adaptive genetic variation in alpine plants is driven by temperature and precipitation. Molecular Ecology 21:3729–3738.

Puritz, J. B., C. M. Hollenbeck, and J. R. Gold. 2014. dDocent: a RADseq, variant-calling pipeline designed for population genomics of non-model organisms. PeerJ 2.

Rellstab, C., F. Gugerli, A. J. Eckert, A. M. Hancock, and R. Holderegger. 2015. A practical guide to environmental association analysis in landscape genomics. Molecular Ecology 24:4348–4370.

Whitlock, M. C., and K. E. Lotterhos. 2015. Reliable Detection of Loci Responsible for Local Adaptation: Inference of a Null Model through Trimming the Distribution of FST. The American Naturalist 186:S24–S36.

Zuur, A. F., E. N. Ieno, and C. S. Elphick. 2010. A protocol for data exploration to avoid common statistical problems: Data exploration. Methods in Ecology and Evolution 1:3–14.
